# Comparison of the pH- and thermally-induced fluctuations of a therapeutic antibody Fab fragment by molecular dynamics simulation

**DOI:** 10.1016/j.csbj.2021.05.005

**Published:** 2021-05-04

**Authors:** Cheng Zhang, Nuria Codina, Jiazhi Tang, Haoran Yu, Nesrine Chakroun, Frank Kozielski, Paul A. Dalby

**Affiliations:** a Department of Biochemical Engineering, University College London, Gordon Street, London WC1E 7JE, United Kingdom; b Department of Pharmaceutical and Biological Chemistry, School of Pharmacy, University College London, 29–39 Brunswick Square, London WC1N 1AX, United Kingdom; c Department of Chemistry, University College London, 20 Gordon Street, London, WC1H 0AJ, United Kingdom

**Keywords:** Antibody fragment, Crystal structure, Protein stability, Protein aggregation, Protein engineering, Molecular dynamics simulations

## Abstract

Successful development of protein therapeutics depends critically on achieving stability under a range of conditions. A deeper understanding of the drivers of instability across different stress conditions, will enable the engineering of more robust protein scaffolds. We compared the impacts of low pH and high temperature stresses on the structure of a humanized antibody fragment (Fab) A33, using atomistic molecular dynamics simulations, using a recent 2.5 Å crystal structure. This revealed that low-pH induced the loss of native contacts in the domain C_L_. By contrast, thermal stress led to 5–7% loss of native contacts in all four domains, and simultaneous loss of >30% of native contacts in the V_L_-V_H_ and C_L_-C_H_ interfaces. This revealed divergent destabilising pathways under the two different stresses. The underlying cause of instability was probed using FoldX and Rosetta mutation analysis, and packing density calculations. These agreed that mutations in the C_L_ domain, and C_L_-C_H_1 interface have the greatest potential for stabilisation of Fab A33. Several key salt bridge losses underpinned the conformational change in C_L_ at low pH, whereas at high temperature, salt bridges became more dynamic, thus contributing to an overall destabilization. Lastly, the unfolding events at the two stress conditions exposed different predicted aggregation-prone regions (APR) to solvent, which would potentially lead to different aggregation mechanisms. Overall, our results identified the early stages of unfolding and stability-limiting regions of Fab A33, and the V_H_ and C_L_ domains as interesting future targets for engineering stability to both pH- and thermal-stresses simultaneously.

## Graphical abstract

**Figure:**
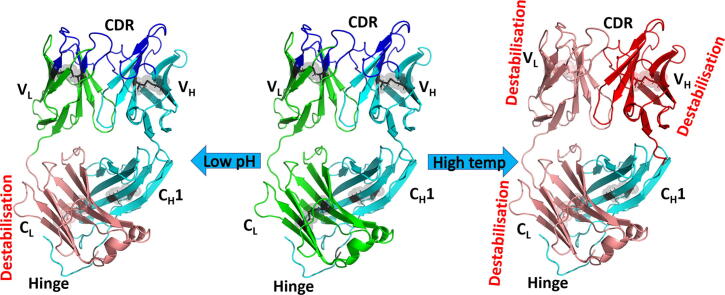

## 1 Introduction

In the last 30 years, monoclonal antibody products have become the main drug class for new approvals in the pharmaceutical industry [1]. To date, over 60 antibody-based drugs are on the market, representing half of the total sales, with over 550 further antibodies in clinical development [2]. They are used as therapeutic drugs to treat human diseases, mainly in oncology, auto-immune diseases and cardiovascular diseases. The use of antibody fragments, such as the antigen-binding antibody fragment (Fab) studied here, brings additional advantages, including deeper tissue penetration due to their smaller size, which has proven beneficial to treat tumors [3]. In addition, Fab fragments lack the Fc domain, and thus are not glycosylated which allows simpler and less costly manufacture due to their expression in prokaryotic systems [4]. However, the lack of the Fc domain leads to their more rapid clearance in humans than for full antibodies.

The stabilization of therapeutic proteins against aggregation remains one of the biggest challenges facing approvals as biopharmaceutical products [5], [6], [7]. Thus, not only their mode of action, but protein stability is a crucial factor to their becoming successful products. Novel antibody products such as Fabs, single-chain variable fragments (ScFvs) and bi-specifics are currently being developed and their properties remain largely unknown. Knowledge about the stability of these pharmaceutical products, especially in the early development stages, would aid in their engineering and the design of antibody fragments that are more aggregation resistant.

Native protein conformations are only marginally stable, and are highly dynamic, hence they are more realistically described as a native ensemble. There is increasing evidence to suggest that under native conditions, aggregation takes place primarily from partially unfolded native-like states [8], [9], [10], [11], [12]. However, little is known about the structures of native conformers that initiate aggregation, or how these are affected by different stress conditions. Local unfolding of proteins can expose aggregation prone-regions (APR), that have the potential to trigger aggregation [13], [14]. APRs are the regions in the protein most likely to form and stabilize the cross β structures that are characteristic of many aggregates, notably hydrophobic sequences with low net charge and a strong β-sheet forming propensity. Generally, APRs are located in the protein core, protected from the solvent, and thus blocked from forming cross-β structure. Under certain stresses, such as an increase in temperature, a change in pH, addition of denaturants, or elevated shear force, structural regions in the protein may destabilize and partially unfold to expose any underlying APRs [15]. Each structural region of the protein can respond differently to stress, and so the overall pattern of responses is likely to vary with each type of stress. Thus, determining the conformational changes that a protein experiences under different stress conditions is important for identifying common routes towards stabilization across all stress conditions via either mutagenesis or formulation.

Molecular dynamics (MD) simulations have been extensively used to study protein stability [16], [17], [18], [19], [20], [21]. MD simulations offer atomic resolution insights into the early conformational events that can take place under different conditions. A conventional way to reflect the solvent pH is to assign the protonation status of chargeable residues during the simulation setting-up stage, based on pKa predictors like Propka [22]. A more recent approach enables “constant pH”, where the protonation status of the protein is constantly updated throughout the simulation [23]. However, this technique is still under refinement, and is currently a computationally expensive “large task” for Gromacs. To date, not many all-atom MD studies on antibody fragments have been reported. MD simulations were used previously to study the aggregation potential of an antibody Fab fragment, from a human IgG1k antibody, via multiple elevated temperature MD simulations at 300 K, 450 K and 500 K [24]. This revealed that domain interfaces deformed prior to the unfolding of individual domains, and that two V_H_-domain sites were potentially labile to aggregation. Their structural deformation increased the solvent-accessible surface area of the APRs in those regions. The unfolding process of an antibody Fab fragment was also studied using an elastic network model, to reveal that the constant regions were more flexible, and unfolded earlier, than the variable regions [25]. MD simulations at 450 K and 500 K have also revealed the stability-limiting regions of an antibody single-chain variable fragment (scFv) [26]. Disruption of the V_L_-V_H_ interface was found to precede the unfolding of the domain structures. In contrast to the studies on the Fab above, the V_H_ domain of the scFv was found to be more thermally resistant than the V_L_ domain.

We previously characterised the stability of A33 Fab [27], [28] at a range of pH, ionic strength and temperatures [12], and revealed a protein that was already close to the high degree of stability required for therapeutic proteins, e.g. to aggregate <1% over 1 year. However, the thermal unfolding transitions (*T*_m_), and aggregation onset (*T*_agg_) temperatures decreased at lower pH, while the aggregation rate (*ln*_agg_) also increased at low pH or high temperature (Table 1). A combination of MD simulation with small-angle x-ray scattering (SAXS) and single-molecule FRET then revealed a deformation in the C_L_ domain that was correlated to increased aggregation kinetics at low pH[12].

**Table 1:** Previous *in vitro* thermal stability and aggregation kinetics for A33 Fab at various pH and incubation temperatures [12]. Fab samples were all 1 mg/ml with ionic strength at 50 mM.

	*T*_m_ (°C)	*T*_agg_ (°C)	*ln*_agg_ at 23 °C (% day^−1^)	*ln*_agg_ at 65 °C (% day^−1^)
pH 3.5	65.3	63.2	−3.0	9.3
pH 4.5	74.9	69.7	−4.1	4.9
pH 7.0	80.0	69.7	−4.7	1.0

**Fig. 1:**
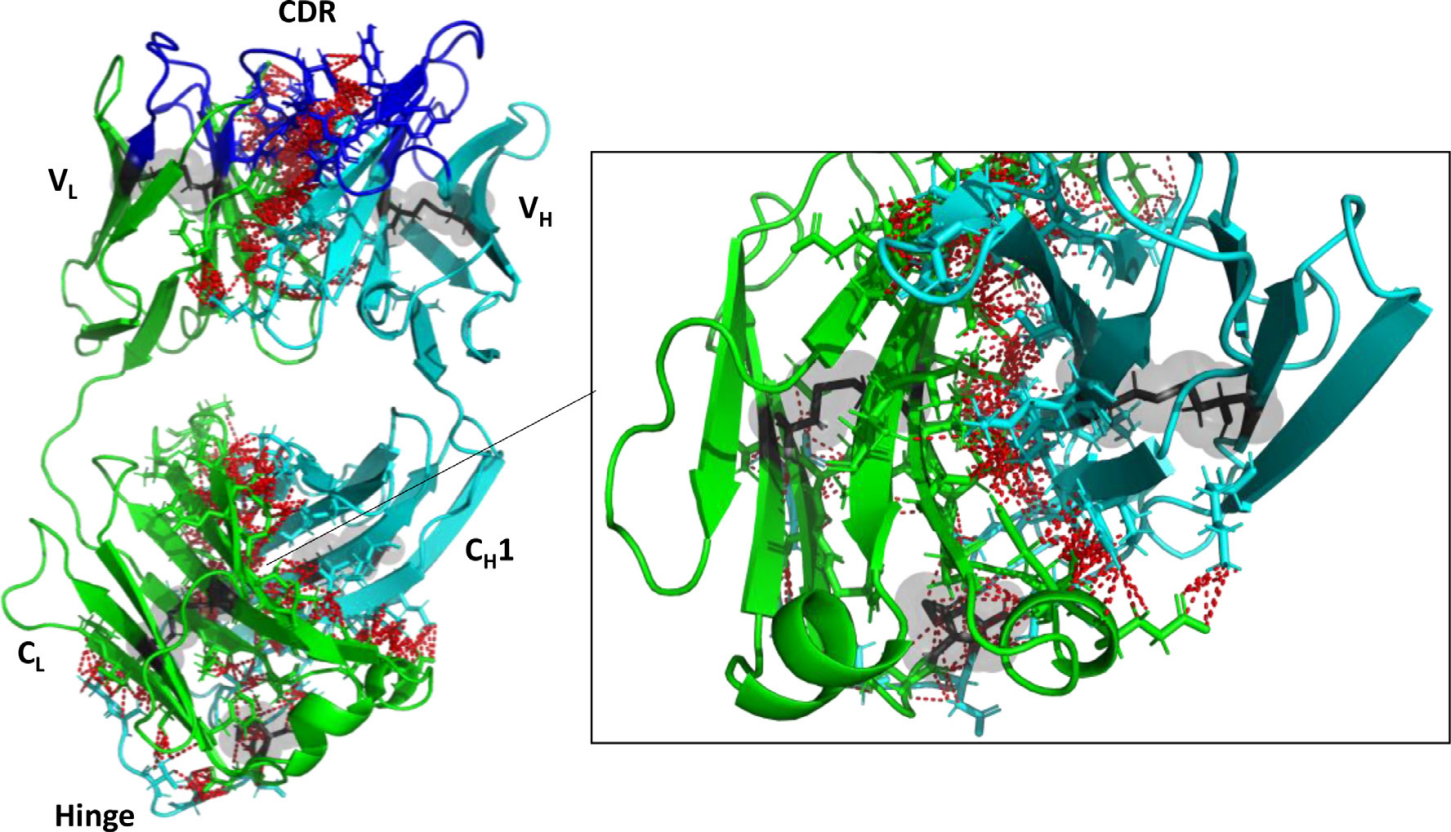
Fab A33 structure with interface contacts highlighted. Fab is composed of light (green) and heavy (cyan) chains. Each chain contains variable (V_L_ and V_H_) and constant (C_L_ and C_H_1) domains. The antigen-binding region at the complementary determining regions (CDRs; blue), are located in the variable domains. There are five disulfide bonds (grey highlights). Contacts between heavy and light chains within 3.5 Å are indicated with red dashed lines arrows. The C_L_-C_H_1 interface is shown up close in the right-inset. (For interpretation of the references to colour in this figure legend, the reader is referred to the web version of this article.)

Fab is composed of one light and one heavy chain (Fig. 1), each comprising a variable (V_L_ or V_H_) and a constant (C_L_ or C_H_1) domain. Each domain forms an immunoglobulin fold, having two layers of β-sheets, an inner β-sheet and an outer β-sheet. The variable domains contain the antigen binding site at their complementary determining regions (CDRs), formed by three loops in V_L_ and three loops in C_L_. There are five disulfide bonds in Fab, four of them intra-domain and the last one between the light and heavy chains at the hinge region. Individual domains interact to form the variable region interface (V_L_-V_H_), and the constant region interface (C_L_-C_H_1). Interface contacts are shown in Fig. 1 and the residues involved in the contacts are listed in SI_Table 1. The variable region interface is mainly formed by aromatic side chains that are tightly packed and located at the centre of the interface (six Tyr, three Phe and two Trp), forming hydrophobic interactions. However, fewer aromatic side-chains are involved in the constant region domain interface (four Phe).

We previously performed MD simulations on a homology model of A33 Fab to generate possible structures that best fit to pH-dependent SAXS data [14], and also to obtain preliminary insights into the early unfolding events at both low pH and high temperature [29]. We have now increased the number of all-atom MD simulation repeats, and more critically, used a recent 2.5 Å resolution crystal structure of A33 Fab (PDB ID 7NFA) [30] as a more accurate starting point to compare the structural dynamics within the Fab domains and chain interfaces, under the two stress conditions of low pH and high temperature. Two different degradation pathways were revealed, leading to partial unfolding of only two regions within the C_L_ domain at low pH, compared to destabilization of all four domains in the high temperature conditions. While conformational deformations at low pH were closely linked to the disruption of glutamate-containing salt-bridges, the high temperature conditions led to a generally increased fluctuation and promiscuity of salt bridge contacts throughout the structure. We examined the impact of these structural fluctuations on the solvent exposure of predicted aggregation-prone regions (APR) under the two stress conditions. Finally, we used *in-silico* mutational prediction tools, and packing density calculations to also reveal the potential of the constant domain interface and C_L_ domain, for engineering that could simultaneously improve stability under both low pH and high temperature conditions.

## 2 Results

### 2.1 RMSD and native contacts of individual domains revealed different unfolding events at low pH and high temperature

**Fig. 2:**
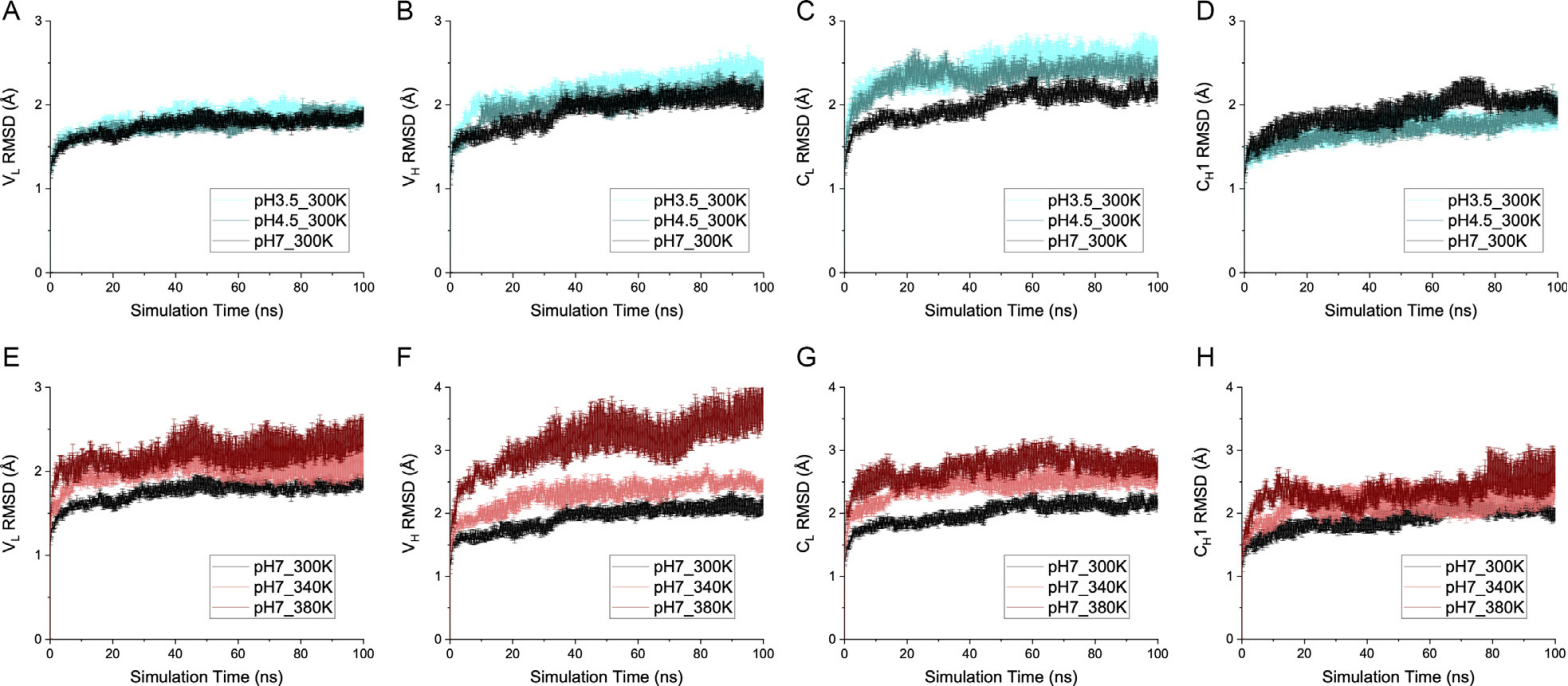
RMSD of individual domains. A, B, C, D) RMSD for domain V_L_, V_H_, C_L_ and C_H_1, respectively, for pH 3.5, pH 4.5 and pH 7 at 300 K. E, F, G, H) RMSD for domain V_L_, V_H_, C_L_ and C_H_1, respectively, for temperature 300 K, 340 K and 380 K at pH 7. In all cases, the average of six independent simulations is shown with the SEM as error.

**Fig. 3:**
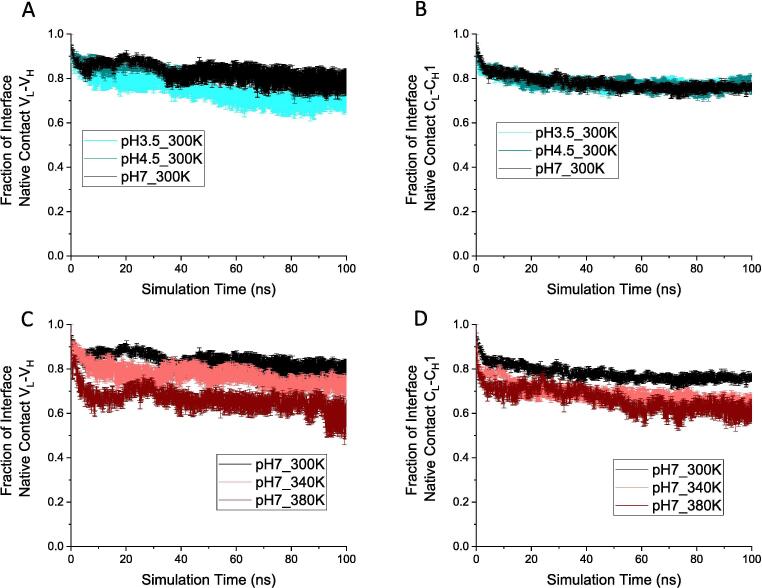
The fraction of interface native contact. A, B) Native contact at V_L_-V_H_ and C_L_-C_H_1 interfaces, respectively, for pH 3.5, pH 4.5 and pH 7 at 300 K. C, D) Native contact at V_L_-V_H_ and C_L_-C_H_1 interfaces, respectively, for temperature 300 K, 340 K and 380 K at pH 7. In all cases, the average of six independent simulations is shown with the SEM as error.

**Fig. 4:**
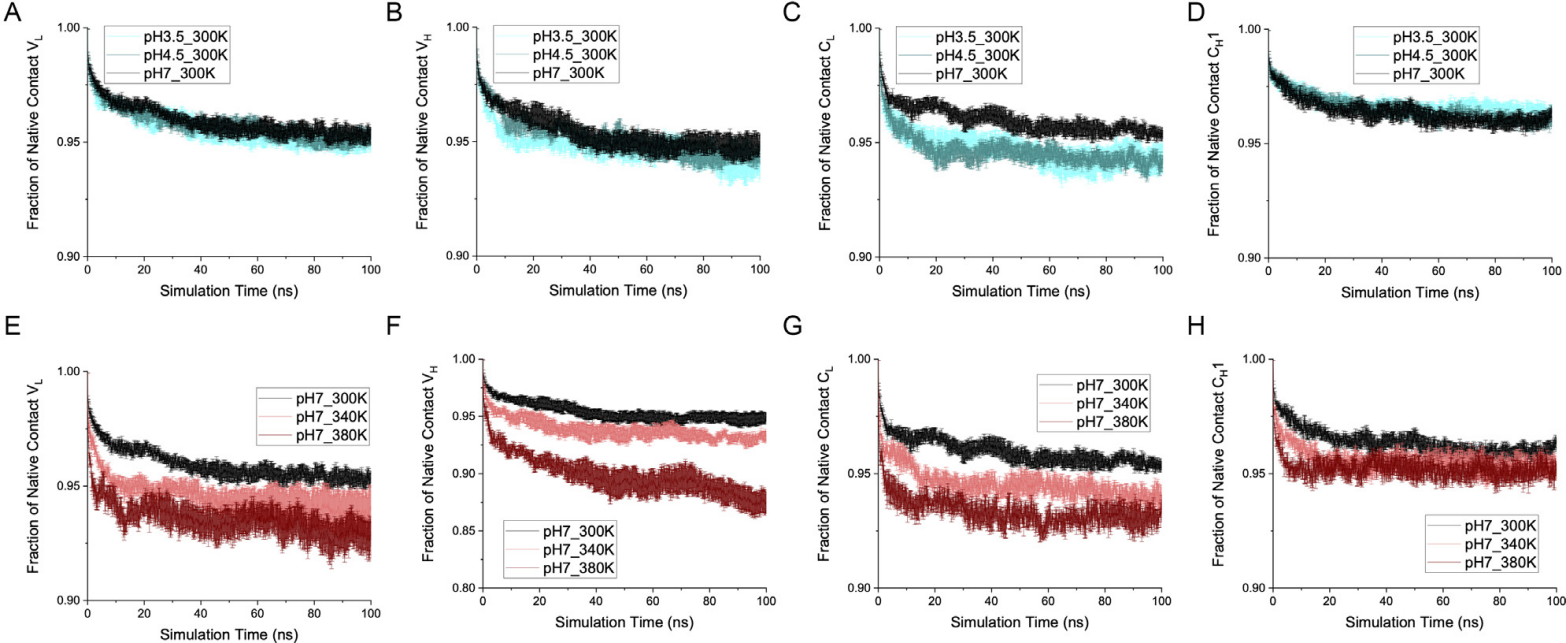
Fraction of native contact at individual domains. A, B, C, D) Fraction of native contact at domain V_L_, V_H_, C_L_ and C_H_1, respectively, for pH 3.5, pH 4.5 and pH 7 at 300 K. E, F, G, H) Fraction of native contact at domain V_L_, V_H_, C_L_ and C_H_1, respectively, for temperature 300 K, 340 K and 380 K at pH 7. In all cases, the average of six independent simulations is shown with the SEM as error.

To determine which domains of Fab A33 are more susceptible to unfolding under low pH and high temperature, we first followed the RMSD of each individually aligned domain (V_L_, V_H_, C_L_ and C_H_1) along the simulations, as changes in RMSD are indicative of a conformational change. Simulations in the unfolding trajectories (pH 3.5 and pH 4.5 at 300 K, for low pH; pH 7.0 at 340 K and 380 K, for high temperature) were compared to the simulations in the native trajectory (pH 7.0 at 300 K). For every condition of pH and temperature, six independent simulations were performed, and their average RMSD for each domain are shown in Fig. 2. We also monitored the fraction of native contacts within each domain, and at the domain interfaces (V_L_-V_H_ and C_L_-C_H_1), during the simulations using a soft-cutoff [31], [32], [33], to understand the temporal relationship between breakage of contacts in each interface, and the unfolding of each domain (Fig. 3 & Fig. 4). The RMSD and radius of gyration (R_g_) of the whole protein are also shown at every condition in SI_Fig. 1; where increased RMSD was observed at the conditions of low pH and high temperature. The trend in R_g_ matches that observed previously by SAXS experiments, although each R_g_ determined by SAXS was fractionally higher than those from MD because solution X-ray scattering is also affected by the hydration shell around the protein [14].

At low pH, the C_L_ domain deformed most significantly while the RMSD of the other three domains remained comparable to those at pH 7, whose trajectory at 300 K is taken to represent a baseline for equilibration into the simulation conditions. The C_L_ RMSD at both pH 3.5 and pH 4.5 deviated notably from that at pH 7, within the first 20 ns of the simulation, then retained a constant difference over the remaining simulation period. At 100 ns, the C_L_ RMSD of pH 3.5 reached 2.58 ± 0.15 Å, compared to 2.18 ± 0.05 Å at pH 7 (Fig. 2C). The domain-based RMSD was also reflected in their corresponding domain-based native contacts (Fig. 4A-D), where only the C_L_ domain saw significant loss of native contacts.

A minor loss of native interfacial contacts in the variable region (V_L_-V_H_) was observed throughout the simulation at pH 3.5, with 69.7 ± 3.5% of native contacts remaining at 100 ns (Fig. 3A) compared to 79.0 ± 5.3% at pH 7. This loss could be attributed to the earlier loss of structure in the V_H_ domain at pH 3.5 than at pH 7. The RMSD of V_H_ increased sharply to 1.96 ± 0.06 Å, in the first 10 ns at pH 3.5, compared to 1.64 ± 0.05 Å at pH 7 (Fig. 2B). Similarly, the fraction of native contacts in V_H_ deviated in the first 15 ns at pH 3.5, to 0.948 ± 0.002, compared to 0.964 ± 0.003 at pH 7 (Fig. 4B). By contrast, almost no difference was observed between the fraction of interfacial native contacts within the constant region (C_L_-C_H_1) of Fab A33, at pH 3.5 and pH 7.0 (Fig. 3B).

The considerable deformation at C_L_ without loss of native contacts at C_L_-C_H_1 interface implies the labile region was not at the C_L_-C_H_1 interface, as will be confirmed and identified with a residue-level analysis discussed below. Considering also the overall increase observed in the calculated global R_g_ at low pH (SI_Fig. 1), the protein appears to undergo considerable partial unfolding at the C_L_ domain, prior to any global unfolding. We also monitored the total number of contacts in the simulations, which included both native and non-native contacts (SI_Fig. 3). While most conditions maintained steady total contacts, the pH 7 condition led to an increase from 650 to 750 contacts during 50–100 ns at the C_L_-C_H_1 interface. This suggests that the protein could naturally rearrange to form more stabilising interactions at pH 7.

The simulations at 300 K were also continued at each pH, until they reached 400 ns (SI_Fig. 2). The C_L_ domain continued to deviate most significantly at low pH, confirming it as the most labile region. The V_L_ domain RSMD at low pH diverged further from that at pH 7, though only slightly, beyond the first 100 ns, suggesting its deformation occurred after that of the C_L_ domain. The V_H_ and C_H_1 domains diverged in RMSD initially, but then converged after 100 ns, implying the heavy chain was less susceptible to destabilisation at low pH. Overall, these findings agreed with previous experimental work, which combined SAXS, atomistic modelling and smFRET to reveal the displacement of the C_L_ domain in Fab A33 at low pH [14].

For thermal denaturation, MD simulations are commonly run at temperatures up to 500 K to attempt to fully denature the protein [19], [24]. Here, we aimed to capture the early thermal unfolding events of Fab A33, which involve only partial unfolding of the protein within the near-native ensemble at equilibrium. For this reason, and to reflect experimental conditions more closely, lower temperatures of 340 K and 380 K were used in our simulations, and compared to those at 300 K. At 300 K, the protein is fully native, while 340 K is close to the *T*_m_ at pH7 and pH 4.5, whereas 380 K is above the *T*_m_ and so denaturing at equilibrium in the physical experiments. Therefore, the 380 K MD simulation would enable us to examine thermal fluctuations under conditions as close as possible to those found in experimental practice, while also accelerating the simulation and leading to more extreme denaturation events expected to occur but with relatively low frequency in native conditions.

The high temperature stress deformed all of the Fab domains, with the V_H_ domain most affected (Fig. 2 E-H). At 380 K, the RMSD of the V_H_ domain rapidly increased to 2.66 Å within 10 ns, and then slowly increased further to 3.79 Å by 100 ns, compared to 1.64 Å and 2.11 Å at 300 K. For the C_L_ domain, the RMSD increased to 2.60 Å after 10 ns, and then more slowly to 2.71 Å after 100 ns at 380 K, compared to 1.79 Å and 2.18 Å at 300 K (all averages within ± 0.2 Å). The V_H_ domain retained much more structure at 340 K (2.46 Å RMSD at 100 ns) than at 380 K, implying it could still resist this more moderate temperature stress.

The native contacts in each domain (Fig. 4E-H) revealed similar trends to the RMSD, with the V_H_ and C_L_ domains losing the most over the simulation. At 100 ns, the V_H_ domain could only preserve 0.875 ± 0.009 fraction of native contacts at 380 K, compared to a fraction of 0.950 ± 0.004 at 300 K. Both the V_L_ and C_L_ domains reserved fractions of 0.92–0.93 at 380 K, which is significantly lower than the fraction of more than 0.95 at 300 K. Apart from the V_H_ domain at 380 K, the native contacts did not see any further reduction after 40 ns, suggesting a temporal equilibrium in the local dynamics on the timescale studied.

Although the native contacts witnessed a considerable drop at the highest temperature for the V_L_, V_H_, C_L_ domains, the absolute difference was still relatively minor (Fig. 4E, G, H), with less than 3% difference in native contacts lost between 380 K and 300 K for the V_L_ and C_L_ domains. While 3% loss of native contacts would not represent a significant loss of global protein stability, it is highly relevant to aggregation from native conditions in which unfolding events are expected to be localised, and relatively rare, yet still trigger protein aggregation. By contrast, more than 20% difference was seen for the interfacial native contacts (Fig. 3C, D), suggesting that the domain interfaces would be lost prior to domain unfolding. At 380 K, there was an average fraction of only 0.598 ± 0.058 contacts in the variable interface and 0.599 ± 0.036 in the constant interface at 100 ns, which were 75–78% of their 300 K counterparts. The C_L_-C_H_1 interface was more susceptible to the elevated temperature stress, with similar loss of native contacts at 340 K and 380 K, whereas at 340 K, the V_L_-V_H_ interface retained 91% of the native contacts observed in the 300 K control. Overall, more contacts were lost in both interfaces at high temperature than at low pH [24]. The total contacts, including native and non-native contacts, could not capture any difference among the various temperatures (SI_Fig. 3), indicating that both the variable and constant domains could rearrange their inter-chain interactions to adapt to the deformation at high temperature. The degrees of deformation measured as RMSD in Fig. 2E-H for each domain, were comparable to the changes in interface native contacts for each pair of domains (Fig. 3C, D), implying that the unstable regions within the domains were closely linked to the light-heavy chain interface.

### 2.2 RMSF and residue-level native contacts unveiled susceptible regions under low-pH and high-temperature stresses

**Fig. 5:**
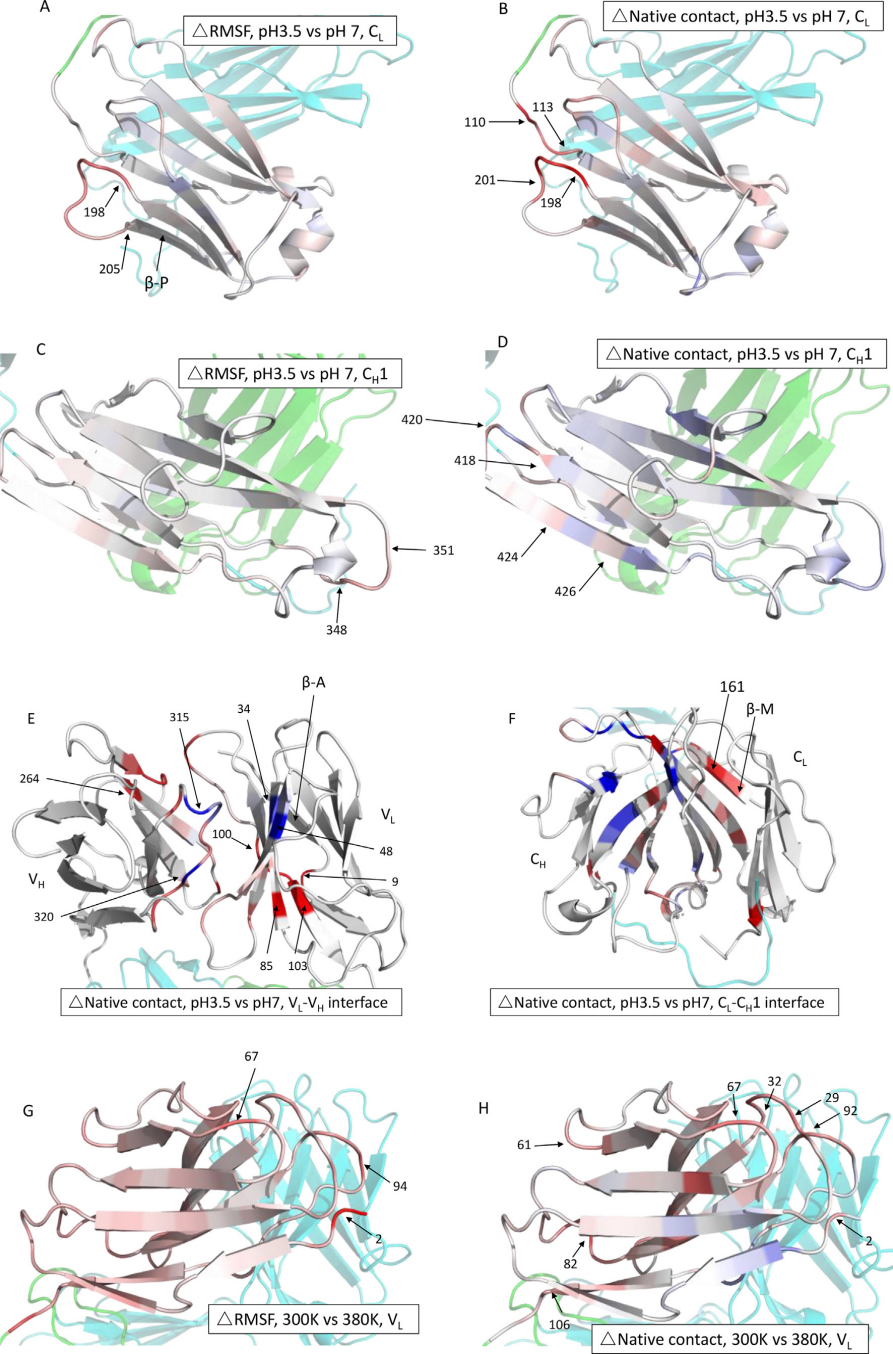

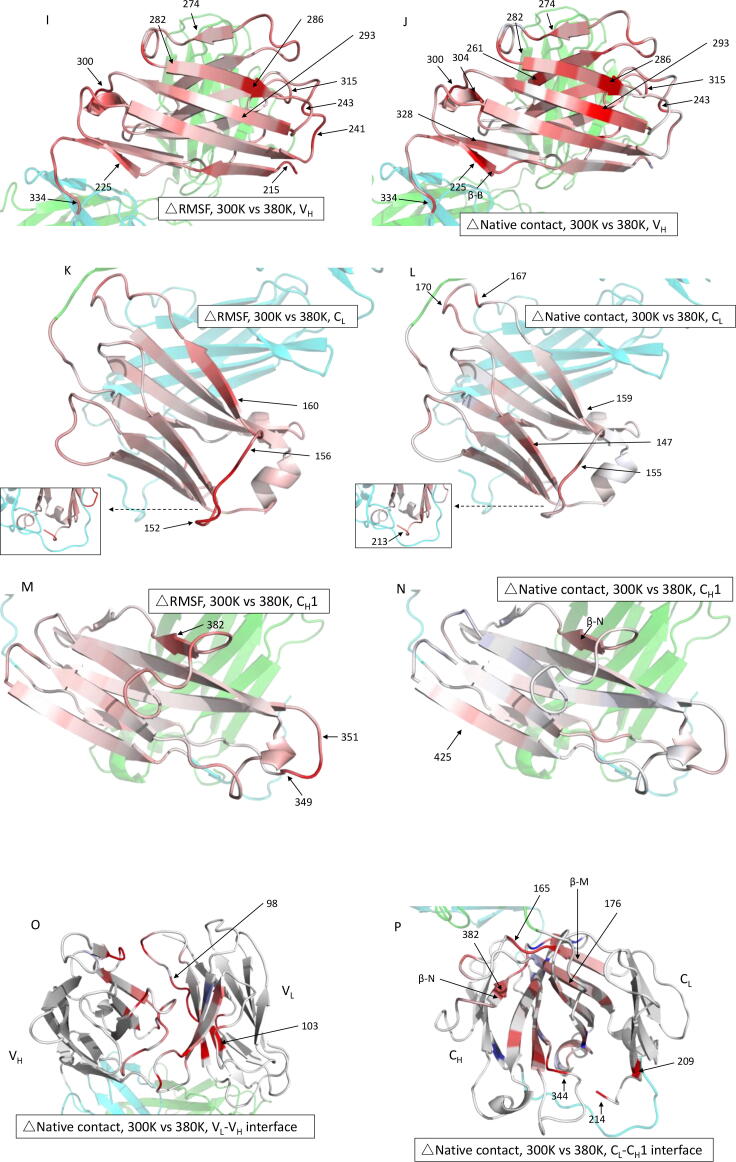
The projection of RMSF and fraction of native contacts on the protein structures. Domains are coloured blue to white to red to represent the difference between pH 3.5 or 380 K versus pH 7, 300 K, for RMSF (-0.15 to 0.15 nm) during 80–90 ns and fraction of native contact (-0.3 to 0.3) at 85 ns. Red indicates increased RMSF or decreased native contact due to stressed conditions (pH 3.5 or 380 K); white indicates no difference between the stressed conditions and pH 7, 300 K. Figures on the left and right are for RMSF and native contact, respectively, with domain name labelled individually. Figure E, F, O, P show the difference of native contact at the light-heavy chain interface. The rest of the structure is partially transparent and coloured in green and cyan for light and heavy chains, respectively. (For interpretation of the references to colour in this figure legend, the reader is referred to the web version of this article.)

To locate the specific residues involved in the loss of native contacts, and structural deformations, under low-pH and high-temperature stresses, the residue-level RMSF and native contacts were analysed. Domain-level structural alignments were made independently prior to RMSF calculations on each domain. This ensured an accurate analysis of residue-level fluctuations without the influence of inter-domain deviations in the structure. The RMSF was calculated for each 10-ns time window, to monitor the progression of flexibility over time. The full dataset showing frames at different times of the simulation are in the SI. The change in RMSF and also the residue-level native contacts at 380 K (compared to 300 K), and at pH 3 (compared to pH 7), each after 85 ns of simulation, are shown as colour changes in the structure images of Fig. 5.

For low pH, the RMSF increased mostly at residues 198–205 in the C_L_ domain (Fig. 5A) and residues 345–351 in the C_H_1 domain (Fig. 5C). At the same time, the native contact fraction decreased by 0.16–0.29 at residues 198–201 (C_L_ domain), compared to that at pH 7 (Fig. 5B). Residues 110–113 also had a significant loss of native contacts at pH 3.5, but with no increase in RMSF (Fig. 5B). Meanwhile, the native contact fraction of residues 345–351 in the C_H_1 domain also did not change despite the increase in flexibility at low pH. A few other individual sites (residues 418, 424, 426) also lost native contacts at low pH (Fig. 5D).

The low pH stress had varied impact on the V_L_-V_H_ and C_L_-C_H_1 interfaces. In the variable interface (Fig. 5E), residues 85 and 100–103 (in V_L_), and several residues in V_H_, had fewer native contacts at pH 3.5. Meanwhile, beta-strand residues 34 and 48 in V_L_, and loop residues 315 and 320 in V_H_, had more native contacts at pH 3.5. In the constant interface (Fig. 5F), the C_L_ domain had fewer native contacts, while the C_H_ domain had more, at pH 3.5 compared to at pH 7.

**Fig. 6:**
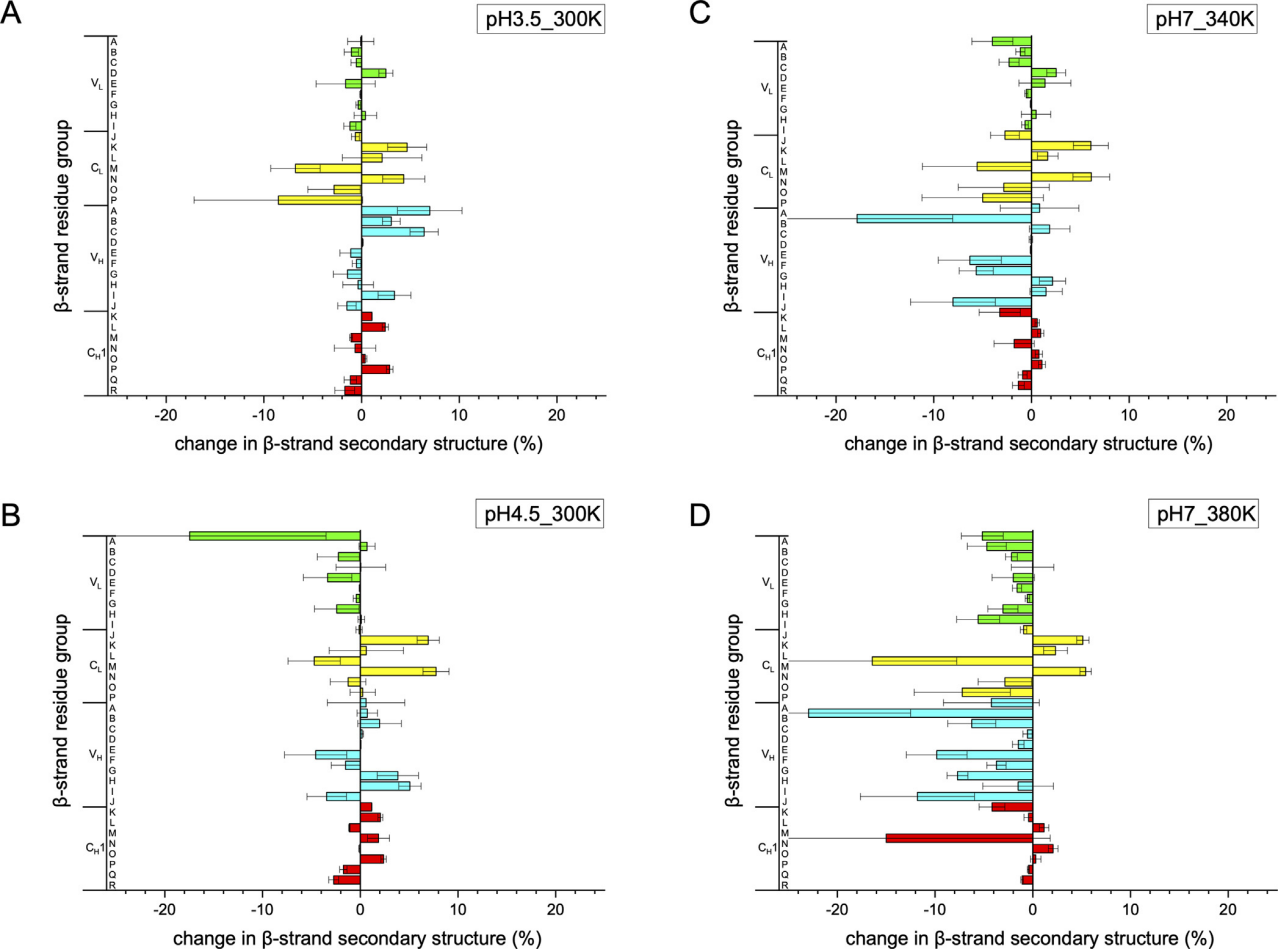
Change of secondary structure for each of the 34 β-strands of Fab A33. Percentage increase/decrease in β-strand secondary structure for each strand in Fab during the simulations, with respect to pH 7.0 and 300 K, for: A) pH 3.5, 300 K, B) pH 4.5, 300 K, C) pH 7, 340 K, D) pH 7, 380 K. Error bars are SEM and are the same and equal for positive and negative values.

The unfolding of individual domains was also analysed by their loss in secondary structure content (Fig. 6A, B). The β-strand P (residues 205–210) in the C_L_ domain at pH 3.5, and β-strand A (residues 4–7) in the V_L_ domain at pH 4.5, each showed a large variability between repeat trajectories. The variability of β-strand P could be associated with the increased flexibility in the loop 198–205 (Fig. 5A); while β-strand A is structurally close to residues 9 and 100–103, where the greatest loss of interfacial native contact occurred (Fig. 5E). A consistent, though relatively small (5–7%) loss in β-strand M (residues 159–163) was also observed at both pH 3.5 and pH 4.5. Its location at the C_L_-C_H_1 interface led to considerable loss of native interface contacts at residues 160–161 (Fig. 5F).

In general, the low pH stress mainly affected the C_L_ domain and resulted in its unique increase in RMSD (Fig. 4C), greater flexibility (RMSF), and loss of native contacts in outer surface loops, and at the C_L_-C_H_1 interface. The remaining majority of interfacial native contacts were comparably affected at both low and neutral pH, which made their total fraction similar during the simulations (Fig. 3A, B).

At high temperatures, the V_H_ domain was the most affected, while the other three domains increased their flexibility in only a few regions (Fig. 5 G-N). The RMSF in V_H_ was relatively unchanged at 340 K, compared to 300 K, but then increased dramatically at 380 K, particularly at residues 215–217, 225–233, 238–246, 265–302, and 329–334 (Fig. 5I, SI). Most of these regions of structure also had significant loss of native contacts at 380 K (Fig. 5J). This thermal stress affected the outer surface and connecting loops of the V_H_ domain more than the inner core.

The other three domains saw increased flexibility overall, but at fewer regions, specifically residues 1–3, 37–42, 91–95 in the V_L_ domain (Fig. 5G, H), residues 150–170 in the C_L_ domain (Fig. 5K, L), and residues 348–353, and 377–382 in the C_H_1 domain (Fig. 5M, N), which had increased RMSF, and loss of native contacts. These events were mainly in loop regions, while the β-strands were more resistant to the thermal stress in these three domains.

At the domain interfaces, most residues had considerably decreased native contact fractions at 380 K compared to at 300 K. This was particularly evident at residues 98–103 of the variable domain interface, and at residues 160–165, 209, 214 and 343–344 of the constant interface, with fractional losses of 0.2 to 0.48. In addition, a cluster of regions located close to the variable domain also had moderate losses in native contact, including residues 174–176 and 382–384.

Upon thermal stress, the loss of β-strand became more persistent, with β-strand reductions in nearly all regions, starting at 340 K (Fig. 6C), and then more pronounced at 380 K (Fig. 6D). Specifically, the V_H_ β-strand B (23%) and C_L_ β-strand M (16%) suffered the most β-strand loss at high temperature. The C, F, G, H, J groups in V_H_ domain also lost 4–12% β-strand at 380 K, and this was partially seen for F, G, J groups at 340 K. Interestingly, the V_H_ J strand was previously found to deform at high temperature in a different Fab[24]. The C_H_1 strand N saw significant loss at 380 K, though not yet at 340 K.

The loss in β-strand content at high temperature was closely linked to the domain deformation (RMSD), elevated flexibility (RMSF), and loss of native contacts. The most evident losses of β-strands, V_H_ B (residue 224–226) (Fig. 5J), C_L_ M (residue 159–163) (Fig. 5P) and that with the highest variability, C_H_1 N (residue 381–383) (Fig. 5N, P), were consistent with the domains that had the greatest losses of native contacts. Strands C_L_ M and C_H_1 N are also located at the constant domain interface, thus contributing to the C_L_-C_H_1 dissociation. The identification of structurally fragile β-strands, namely C_L_ strand M at low pH, and most V_H_ β-strands at high temperature, was consistent with the instability observed from the domain-based RMSD and native contacts. We also identified strands K and N in the C_L_ domain to be surprisingly intact in all four stress simulation conditions. These connect the inner and outer C_L_ domain and were not directly involved in the observed contact losses. While the prevalence of these two β-strands may be conformationally stabilising under acidic and thermal stresses, as discussed further below, their rigidity and predicted role as aggregation prone regions (APRs) may also drive the experimentally observed formation of protein aggregates and fibrils under these conditions (see the APR discussion below).

### 2.3 Salt bridge analysis identifies key stabilizing salt bridges

**Fig. 7:**
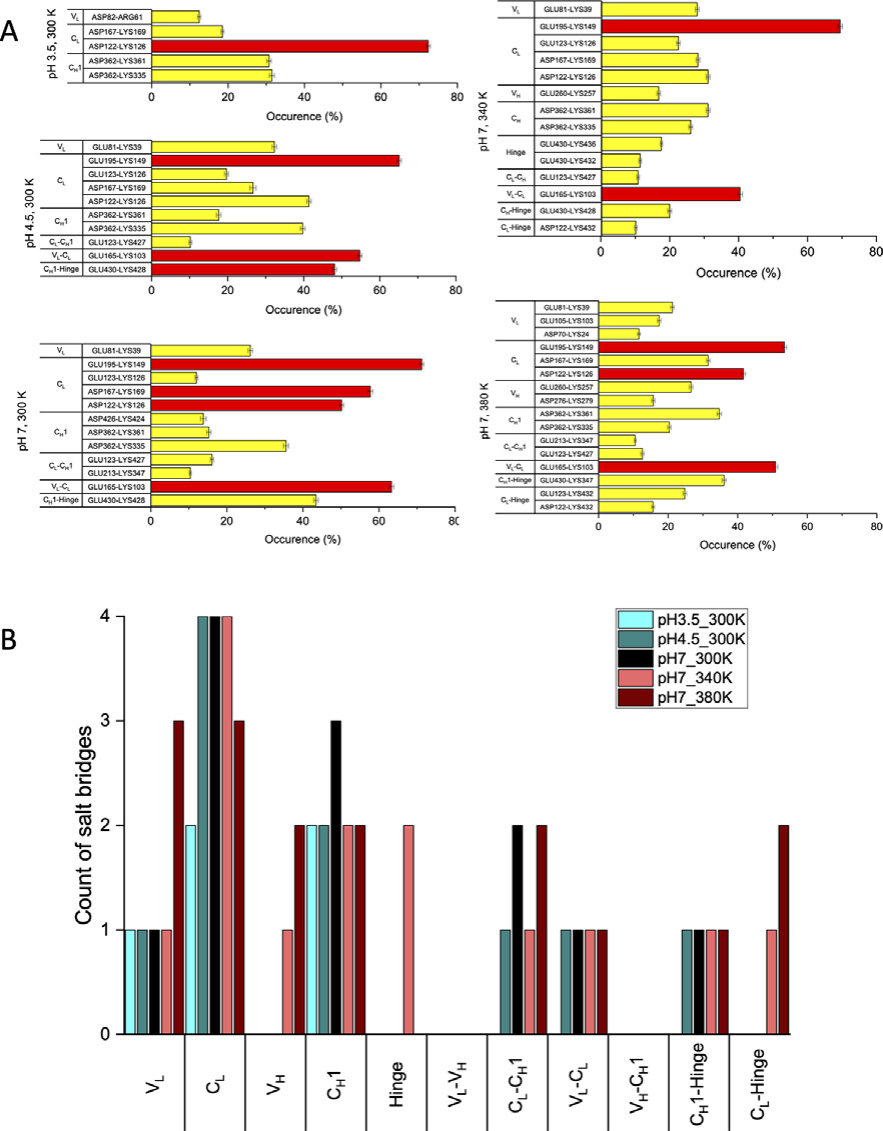
Salt bridge analysis. (A) Occurrence of salt bridges during simulations, above a 10% threshold. Values shown are the average of six independent simulations with error bars to show their SEM. (B) The total salt bridge count with occurrence over 10% for each domain or domain interfaces, and under each condition simulated.

To identify the ionizable residues that potentially drive the conformational changes at low pH and high temperature, a salt bridge analysis was performed. Salt bridges were identified over the simulation time for all the MD simulations carried out, using an O-N bond distance cutoff of 3.2 Å. From these, the occurrence (%) of each salt bridge during the simulation was calculated and averaged for the six independent repeats at each condition. The salt bridges with occurrence over 10% are listed Fig. 7 (A). The most persistent ones are highlighted in the structure, and a complete dataset with all salt bridge occurrences is shown in the SI_Fig. 5, for each condition throughout the simulation period. The count of salt bridges at various domains or domain interfaces are shown in Fig. 7 (B).

At pH 7.0, 300 K, a total of 12 salt bridges were present to at least a 10% occurrence. Interestingly, Asp362 paired with both Lys335 and Lys361. This is consistent with previous work, which found that salt bridges break and reform, and not always with the same partner [34]. The most persistent (as occurrence %) salt bridges at pH 7.0 were Glu195-Lys149 (71.2 ± 0.5%), Glu165-Lys103 (63.3 ± 0.6%), Asp167-Lys169 (57.7 ± 0.7%), Asp122-Lys126 (50.2 ± 0.5%), and Glu430-Lys428 (43.4 ± 0.6%). The top four salt bridges were all located in the C_L_ domain. As a result, the C_L_ domain had the largest number of salt bridges, with four intra-domain ones, two paired with the C_H_1 domain and one paired with the V_L_ domain (Fig. 7B). C_H_1 and V_L_ had three and one intra-domain salt bridges, respectively, while the V_H_ domain did not contain any at pH 7.0, 300 K. In fact, no salt bridge was associated with the V_H_ domain for more than 10% occurrence.

When the pH decreased to pH 4.5, two salt bridges were lost (at >10% occurrence) compared to at pH 7.0. Salt bridges Glu195-Lys149, Glu165-Lys103, Glu430-Lys428, Asp122-Lys126 remained the most popular at pH 4.5, but with their occurrence reduced by up to 18% compared to at pH 7.0. The Asp167-Lys169 salt bridge had the greatest decrease in occurrence at pH 4.5, falling to less than half that at pH 7.0, suggesting that it could have a critical impact under the low pH stress.

The further decrease to pH 3.5, resulted in a significant loss of salt bridges and only five in total. All the salt bridges involving Glu were lost, including three of the most frequent at pH 7.0. Only pairings of Asp with Lys or Arg remained, consistent with the average pKa of 4.25 and 3.65 for Glu and Asp, respectively [35]. Critically, the salt bridges involving Glu often occurred over long-distances in primary sequence, whereas those with Asp were mostly paired locally in sequence. Thus, at low pH the Glu-containing salt bridges were also most likely to impact on protein unfolding.

As half of the salt bridges were associated with C_L_ domain, this domain was also most prone to the low pH stress (Fig. 7B). At pH 3.5, all three inter-domain salt bridges for C_L_ were lost, and only two of four intra-domain salt bridges remained. Loss of these salt bridges at low pH, would therefore substantially destabilise the C_L_ domain, and promote the observed C_L_ domain displacement.

### 2.4 High temperature led to more salt bridges, but with lower occurrence, reflecting an increased conformational flexibility

At pH 7.0, a total of 14 and 16 salt bridges were observed at 340 K and 380 K, respectively, compared to 12 at 300 K. Most of the salt bridges found at 300 K, were retained at 340 and 380 K. Glu195-Lys149, Glu165-Lys103 and Asp122-Lys126 continued to be the most frequently formed salt bridges, while nearly two thirds of the salt bridges had an occurrence of <20%. The salt bridges experienced more frequent disruption and reformation at the elevated temperatures. Moreover, several ionisable residues did not always pair with the same partner, including E430, E123, D362, and D122, reflecting the increasing conformational flexibility and instability. The V_H_ domain, which had the greatest structural deformation, formed 1–2 new salt bridges to 16–27% occupancy. While the elevated temperature led to the transient sampling of a wider range of salt bridges, through increased conformational flexibility, this is likely to provide a diminishing degree of stability to the native Fab ensemble.

### 2.5 Packing density and solvent accessibility reveal suboptimal packing at the C_L_-C_H_ interface

**Fig. 8:**
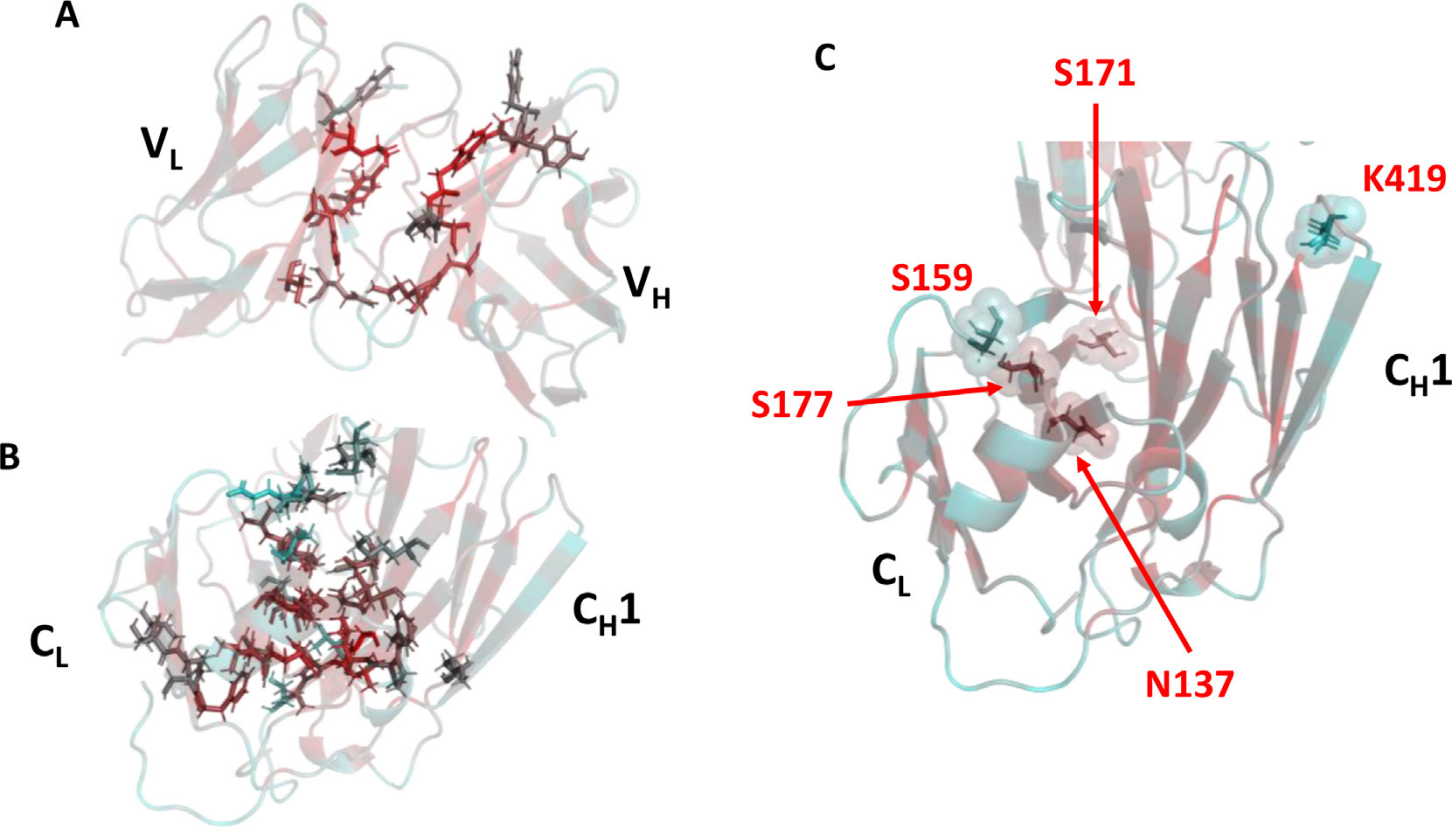
Calculated packing densities of all residues in the Fab A33 crystal structure. Occluded surface packing (OSP) values are shown for the A) variable and B) constant domains. High packing values are shown in red and low values in cyan. Residues in the β-strands within the V_L_-V_H_ and C_L_-C_H_1 domain interfaces are highlighted as sticks. C) Residues identified by FoldX and Rosetta that could be stabilised further are highlighted as spheres. (For interpretation of the references to colour in this figure legend, the reader is referred to the web version of this article.)

The packing density of each Fab A33 residue was calculated using the package occluded surface (OS) software, which calculates occluded surface and atomic packing [36], [37]. The occluded surface packing (OSP) value of each atom is calculated from normal vectors that extend outward from the atom surface until they intersect a neighboring van der Waals surface (SI_Fig. 6). This value is 0.0 for completely exposed residues and 1.0 where 100% of molecular surface is in contact with other van der Waals surfaces. There are 16 and 39 β-sheet residues involved in the V_L_-V_H_ and C_L_-C_H_ interface contacts, respectively (SI_Table 2). The fewer contact residues in the V_L_-V_H_ interface may provide more flexibility to allow antigen binding. The OSP values for V_L_-V_H_ and C_L_-C_H_ interfaces are mapped onto the Fab structure in Fig. 8. V_L_-V_H_ interface residues had more optimal packing than in the C_L_-C_H_ interface despite the fewer number of contacts, with average OSP values of 0.488–0.515, and 0.405–0.410, respectively (SI_Table 2). While the relative rigidity of β-strand residues contributes to the stability of the Fab inner core, their suboptimal packing density reveals considerable room for improvement, such as introducing more hydrophobic interactions that stabilise the Fab inter-domain core. The Solvent Accessible Surface Area (SASA) for each residue of the Fab crystal structure was also calculated and mapped onto the structure (SI_Fig. 8). This confirmed that several interface residues were not solvent accessible, and yet had relatively low OSP values, implying they were not well packed by their surrounding residues (SI_Fig. 9).

### 2.6 FoldX and Rosetta predict potential stabilising mutations

**Fig. 9:**
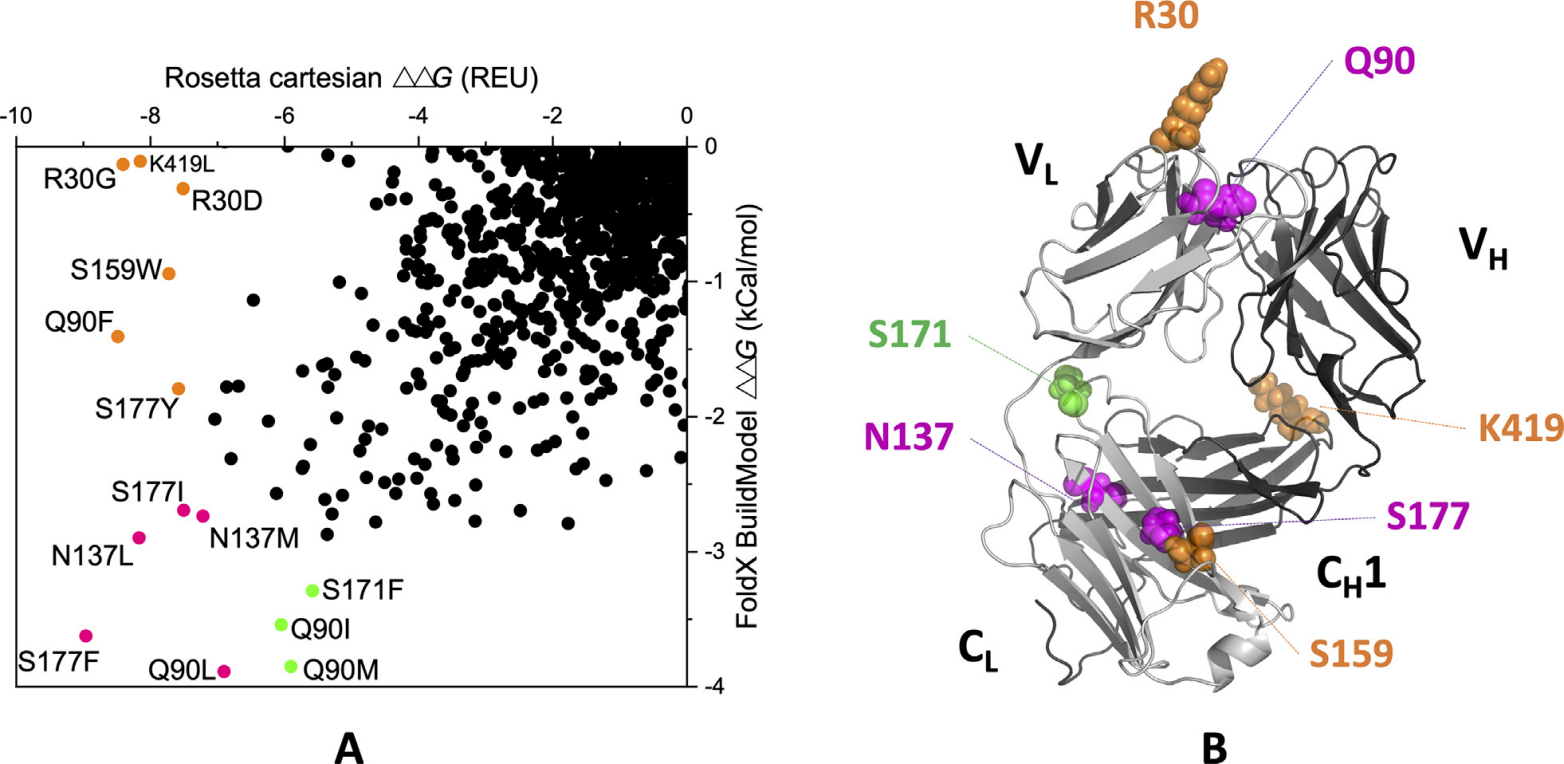
Distribution of Fab-stabilizing mutations predicted by FoldX and Rosetta. A) Correlation between FoldX and Rosetta predictions. Mutations predicted to be most stabilizing by both tools are highlighted magenta. Mutations predicted be most stabilizing by FoldX only are in green and those by Rosetta only in yellow. B) Top predicted stabilising mutations mapped to Fab A33 structure, following the same colour scheme as in A). (For interpretation of the references to colour in this figure legend, the reader is referred to the web version of this article.)

To explore the repacking of the domain interfaces, computational tools were applied to design stabilising mutations. Protein modelling software such as FoldX [38], [39] and Rosetta [40], [41] predict the relative changes in folding free energy (ΔΔ*G*) between the Gibbs free energies (Δ*G*) of the protein carrying a simulated point mutation and the wild-type protein, to find those mutations that will most significantly reduce the free energy of the protein. These approaches are often also combined to find consensus predictions [42], [43]. For Fab A33, we calculated the ΔΔ*G* from both FoldX and Rosetta, for all possible single-mutant variants when accessing all 19 substitutions across the 442 residue positions in Fab A33, totaling 8398 mutations. FoldX identified 1612 (19.2%) of these mutations as stabilizing, while Rosetta-ddG identified 1606 (19.1%). Of these, 852 (10.1%) were predicted by both algorithms. Fig. 9A shows the correlation between the ΔΔ*G* values for stabilising mutations as predicted by both FoldX and Rosetta, highlighting 25 stabilizing mutations ranked at the top by one or both algorithms. The location of mutations involved in the interface contacts were also highlighted in the SI_Fig. 10, and were found to be distributed evenly within the correlation plot for all 8398 variants.

Five mutations were highlighted in magenta in Fig. 9A and B, as those predicted by both algorithms to have the greatest potential for stabilization. These mutations were in residues S177, N137, and Q90, and their most stabilizing substitutions were to more hydrophobic amino acids, such as Phe, Leu, Ile, Tyr and Trp (SI). S177 and N137 are located in the constant domain interface (Fig. 9B), where the packing density was low as previously discussed. While N137 had a relatively good OSP value of 0.479, the N137L and N137M mutations led to higher OSP values of 0.528 and 0.541 (SI), respectively. S177 was not directly involved in the interface contacts (SI), and the suggested mutations S177F/I/Y did not introduce new contacts with the C_H_1 domain (SI files), although their OSP values increased from 0.438 (S) to 0.510–0.513. Thus, any stabilisation for the S177 mutations would be through better packing within the C_L_ domain.

Other predicted mutations within C_L_ included S159 and S171. S159 is at the C_L_-C_H_1 interface in C_L_ strand M but again S159W could not introduce new contacts with C_H_1 domain (SI). Loop residue S171 interacts with the switch loop connecting the V_L_ and C_L_ domains. Thus overall, the C_L_ domain has a relatively high potential for stabilization, through repacking of the C_L_-C_H_1 interface (N137L/M), within the C_L_ domain (S177F/I/Y, S159W), or through improved interaction between C_L_ and V_L_ (S171F) (Fig. 8C). These could enhance the more labile structural regions observed in the MD simulations, such as the deformation of C_L_ at low pH and the dissociation of the C_L_-C_H_ interface at high temperature.

In addition to the mutations in the C_L_ domain, several other mutations were recommended (Fig. 9). Residue Q90 at the end of β-strand H (Fig. 6A), was suggested by both Rosetta and FoldX, for mutations to aliphatic (Q90L, Q90I), non-polar (Q90M) and aromatic (Q90F) side-chains. The Gln90 side-chain interacts with strand C within the V_L_ domain and so the various hydrophobic mutations suggested could improve the V_L_ domain stability. Residue R30 in the CDR of the V_L_ domain, was suggested by Rosetta to be mutated into Gly or Asp. These were surprising as Gly might increase CDR loop flexibility [44], while the Asp substitution would not disrupt or form new salt bridges as examined by VMD. In any case, they would not be good candidates for general framework stabilisation due to the CDR role in antigen binding.

### 2.7 Solvent exposure of different aggregation-prone regions promotes different aggregation pathways for low pH and high temperature

The aggregation pathways of Fab A33 at low pH and high temperature at pH 7.0, are already known to result in different aggregate morphologies [12]. Here we explored whether the two conditions also exposed different aggregation-prone regions (APRs). APRs can be predicted from sequence information, and either assume a fully unfolded protein, or otherwise refine the prediction by factoring solvent exposure of the APR based on structure and dynamics information. The sequence-based predictions are based on either the intrinsic properties of amino acids, or their compatibility with protein structural features in known amyloid fibril structures. Examples of sequence-based predictors include PASTA 2.0 [45], TANGO [46], AGGRESCAN [47], MetAmyl [48], FoldAmyloid [49] and Waltz [50]. As the ability of APRs to trigger aggregation depends upon their solvent accessibility, more recent structure-based predictors consider the three-dimensional structure of the protein and in some cases also their potential modes of partial unfolding. Examples include AGGRESCAN 3D [51], AggScore [52], SAP [53] and Solubis [54]. Here, we want to compare the solvent accessibility of APRs in Fab A33, between our MD simulations at the unfolding conditions and at the reference trajectory. Thus, we used sequence-based APR predictors to determine the APRs in Fab A33, and then determined their solvent accessible surface area (SASA) from the MD simulations, for relative comparisons.

**Fig. 10:**
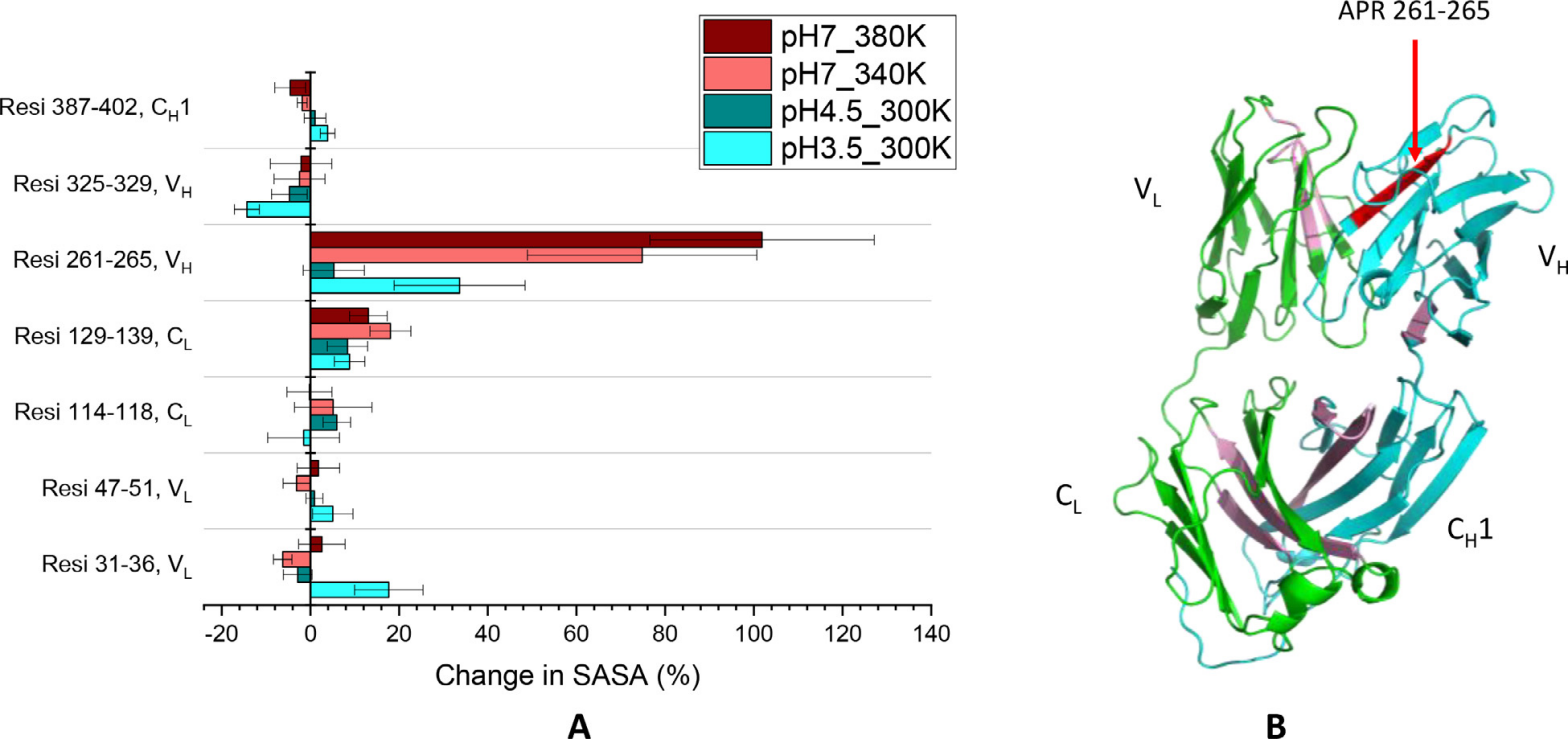
The APRs’ SASA change in simulation and their locations. A) Change of SASA for each of the 7 APRs of Fab A33. Percentage increase/decrease during the simulations was shown with respect to pH 7.0, 300 K. Error bars are SEM and are equal for positive and negative values. B) The locations of the predicted APRs. The light chain and heavy chain are coloured in green and cyan, respectively. The seven APRs are coloured in pink except the APR 251–256 in red. (For interpretation of the references to colour in this figure legend, the reader is referred to the web version of this article.)

We used four sequence-based predictors to determine the APRs in Fab A33, PASTA 2.0, TANGO, AGGRESCAN and MetAmyl. APRs were selected when three out of the four predictors identified an aggregation-prone sequence (SI). Seven APRs were found, namely residues 31–36, 47–51, 114–118 and 129–139 in the light chain and residues 261–265, 325–329 and 387–402 in the heavy chain. The presence of these APRs was confirmed using Amylpred2 [55], a consensus tool of eleven existing algorithms (SI). All seven APRs were located in the interior of Fab A33, and thus protected from the solvent (Fig. 10B). Exposure of any one of these APRs as a result of a conformational change by an environmental stress, has the potential to trigger aggregation. Thus, the SASA of each APR during the simulations was calculated, to give the difference in solvent accessibility between stressed conditions and the reference simulation, as shown in Fig. 10A. APR 261–265 had the most striking increase in solvent exposure, with a 75–100% SASA increase at 340–380 K and a 34% increase at pH 3.5. The elevated temperature conditions also gave small increases in SASA for APR 129–139 (12–18%). The low pH condition gave a modest increase for APR 129–139 (8%), but also an increase of 18% for APR 31–36, and a decrease of 14% for APR 325–329. The other APRs did not have significant changes compared to the reference condition. Thus, while at elevated temperature, the APR exposure was dominated by APR 261–265, at low pH it was additionally closely matched by the increase in APR 31–36 exposure. These differences could potentially influence the final morphology observed experimentally [12].

Previously, the SAXS solution structures at pH 7 and pH 3.5 showed a 3% increase in SASA for APR 387–402, and yet few changes at other sites [14]. Thus, while the same 3–4% change at APR 387–402 was observed in the current MD simulations, other APRs had more significant increases in SASA at low pH. This difference may reflect the fact that the SAXS structure analysed was based on a single MD frame that best fitted the experimental data for the average ensembles, the MD trajectory analysis we have done examines the entire collection of states within the ensembles.

APR 261–265 is within the V_H_ domain, and its significant solvent exposure could be a source of instability to aggregation at both elevated temperature and decreased pH. The increased solvent exposure arose through the loss of beta-sheet content in V_H_, while the APR itself was in β-strand E, which did not see significant loss of secondary structure (Fig. 6). APR 129–139, which had the second largest exposure, corresponds to β-strand K of light chain. Similarly, it is one of the two strands that uniquely remained intact in both high temperature and low pH stresses (Fig. 6). APR 129–139 was also reported to display increased hydrogen–deuterium exchange for Bevacizumab aggregates incubated at 70 °C compared to native monomers [56]. Thus, the exposed β-sheets completely remained or even increased their secondary structure, which would make them strong contenders to act as precursors for the formation of amyloid fibrils.

For the low pH stress, the increased APR exposure at sites within V_H_, V_L_ and C_L_ could potentially all contribute to aggregation instability. A previous analysis of APR solvent accessibility in solution structures obtained by small-angle X-ray scattering at neutral and low pH [14], revealed the C_L_ domain to be critical at low pH. However, the two APRs in the C_L_ domain led to only modest increases in SASA in our simulation results. Potentially, the structural changes in the C_L_ domain at low pH could also trigger the exposure of the two APRs in the V_H_ domain, leading to multiple routes to aggregation.

### 2.8 Comparison to natural sequence variations in Fabs

**Fig. 11:**
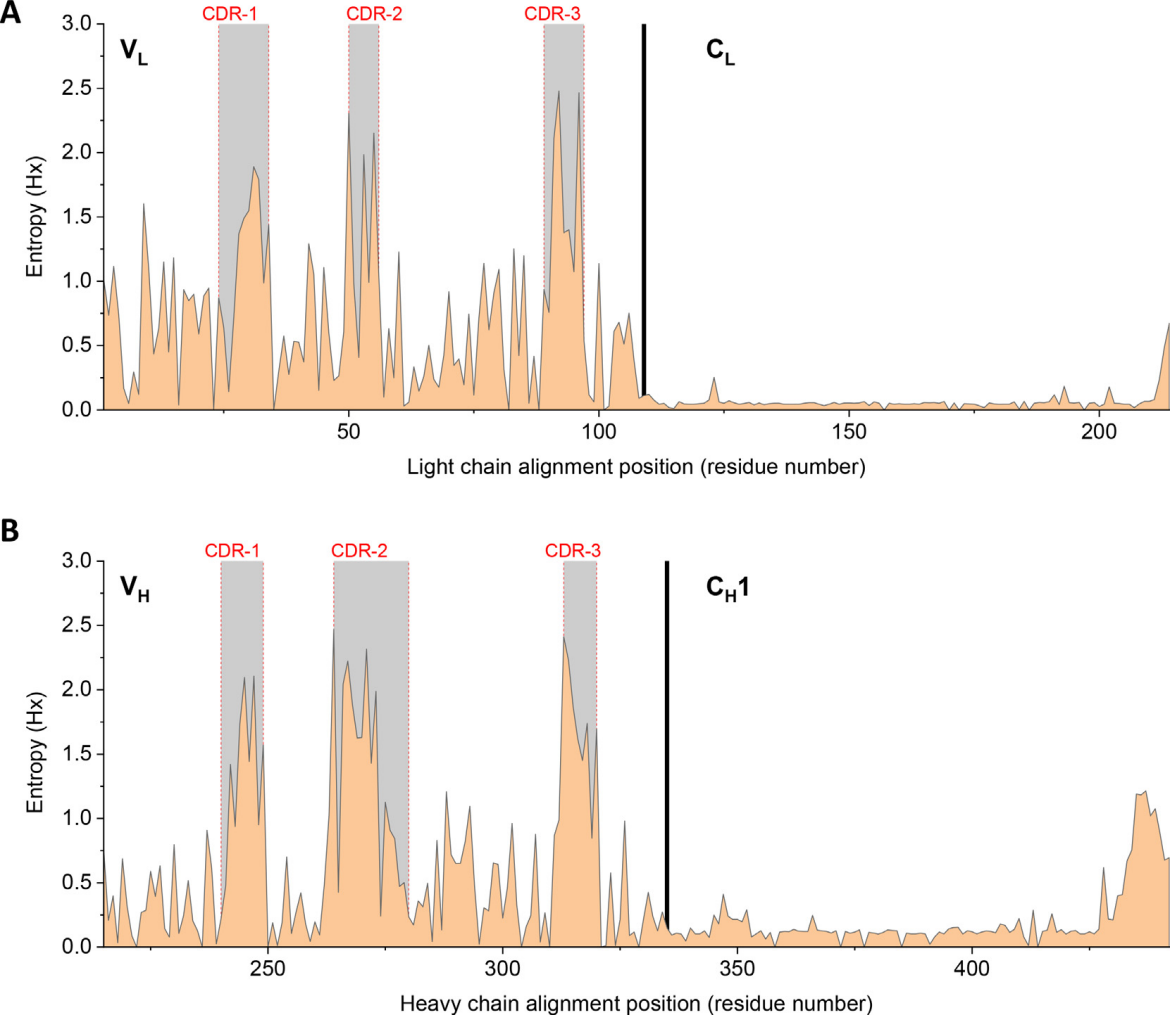
Sequence entropy of Fab sequences. The Fab A33 light and heavy chain sequences were aligned with their homologous sequences and entropy calculated by BioEdit[60]. (A) Entropy (Hx) of 1170 Fab light chains. (B) Entropy (Hx) of 613 Fab heavy chains. Light and heavy chains are from human and mouse species. Variable and constant domains are annotated and separated by a solid vertical line. CDRs are masked in grey.

To understand the extent to which our findings with Fab A33 may be applicable to other Fab structures, we analysed the sequence entropy across homologous sequences, as shown in Fig. 11, and also visualised the consensus residues using sequence logo representations[57], [58] (SI). It was not surprising to observe greater variability in the two variable domains, especially the hypervariability of the six CDRs [59], compared to the constant domains. The hinge region also had a significant entropy rise, reflecting the increased tolerance to variations expected in connecting loops. Most other positions were predominantly occupied by one residue, with some occasional natural variations, but mostly limited to substitutions with similar properties, such as D/E, Q/E, K/R.

Herein, we specifically investigated the degree of sequence conservation within the unstable regions under low pH or high temperature stresses. At low pH, the unstable regions mostly located at the C_L_ domain which exhibited very low entropy scores of *ca.* 0.05 (Fig. 11), including residues 4–7 (β-strand A), 100–103, 110–113, 159–163 (β-strand M), 198–205, 205–210 (β-strand P) and 345–351. Therefore, the low pH stress observed in this study is highly likely to play a similarly destabilising role in the other antibodies of the same class. At high temperature, the V_H_ domain was the most affected, including several flexible regions associated with the CDR regions which thus exhibited high sequence entropy, including residues 238–246, 265–302, and 91–95. Meanwhile, the two domain interfaces were also affected by high temperature. As a result, the impact of high temperatures could vary significantly across the Fab sequence family, although with common issues in the C_L_-C_H_1 interface.

The mutations suggested by FoldX and Rosetta were typically from polar to more hydrophobic residues (Fig. 9), and most of them were not observed in natural variants. Few mutations were at sites of high entropy, such as for R30, for which the mutations R30D and R30G ranked only as the 4th and 6th most common among natural variations. None of the four Q90 mutations, Q90L/I/M/F, appeared in the natural variations. Thus, while the engineering strategy offered by FoldX and Rosetta would aim to stabilise the protein *in vitro* as required by therapeutic product formulators, similar mutations may not have arisen naturally as they are not necessarily compatible with the many additional selection pressures subjected to natural variants *in vivo* as part of whole antibodies and the adaptive immune response.

## 3 Discussion

Antibody-based products are the main class of approved biopharmaceuticals, due to their high target specificity [1]. However, there are many barriers to their successful development into therapeutics, with protein aggregation being perhaps the most common and challenging to prevent [5]. There is a need to identify potential instabilities of therapeutic proteins during their early development, particularly against stresses that they will encounter during manufacture, storage and delivery. This would allow their early elimination from further development, or otherwise rational mutagenesis into more stable products. In this context, we have elucidated the first unfolding events that take place on a humanized Fab A33 using atomistic MD simulations, and compared these to predictions of potentially stabilising mutations using computational tools.

Our simulations showed that the high temperature stress led to loss of native structure in all domains except C_H_1, and in the domain interfaces. By contrast, only the C_L_ domain was significantly affected at low pH stress, revealing different unfolding pathways for the two denaturing stresses. Two loops on the external surface of the C_L_ domain were particularly affected low pH, yet while retaining an intact C_L_-C_H_1 interface. Salt bridges were particularly critical to loss of stability at the low pH stress. High temperatures led to increased flexibility across the whole structure, causing considerable loss of native contacts and secondary structure, most noticeably in the V_H_ domain and V_L_-V_H_, C_L_-C_H_1 interfaces.

Packing density, FoldX and Rosetta analyses revealed under packed residues located in the interface between domains, with significant potential for stabilization through mutagenesis, notably in the C_L_ domain and constant domain interface. Mutations outside the C_L_ domain were also identified, including with the potential to rigidify the V_L_ domain, although this may impact the dynamics and binding via the CDRs.

In order to gain insights into the mechanisms by which aggregation might occur, APRs in the interior of Fab A33 were identified, and their solvent accessibilities compared. The exposure of of one APR in the V_H_ domain was linked to instability in that domain at elevated temperature, and more modestly at low pH. At low pH, an indirect causality may exist between local unfolding in the C_L_ domain, and exposure of additional APRs in the variable domains. These results highlight the importance of identifying the underlying causes of protein instability under the different stresses it might encounter. It also provides insights into the stability and robustness of the therapeutically relevant Fab A33, and offers a potential route for the engineering and design of a more aggregation resistant antibody fragment.

## 4 Materials and methods

### 4.1 Fab A33 crystal model

Several residues were not resolved in the original crystal structure [61], including residue 214 in the light chain and residues 215, 315, 346–351 and 432–442 in the heavy chain. Since a full-length PDB is required in molecular modelling and simulation, Rosetta [62] (rosetta/2018.48.60516-mpi) was used to fill the missing residues with energy minimised, and validated with our previous *in vitro* stability data. The UCL high-performance computing facility was used to accelerate the modelling work. Both the raw crystal and full-length structures are deposited online. Readers are welcomed to use other methods to pack the missing residues.

The “RosettaCM”[63] application was used to fill the missing residues. The raw crystal structure was used as the template structure. The full-length Fab sequence was aligned to the template sequence at https://www.ebi.ac.uk/Tools/msa/clustalo/, using “-“ as missing residues, and saved in a .aln file. 3-residue and 9-residue fragment files were obtained from robetta.bakerlab.org/fragmentsubmit.jsp The .aln alignment file was further converted to Grishin format. Afterwards, the full-length sequence was threaded onto the template sequence, and “RosettaCM hybridize” was performed to generate more than 40,000 structures. The structure with lowest energy and five intact disulfide bonds was further energy minimised using Rosetta “relax” [64], [65], [66], [67] application (SI), resulting more than 20,000 conformations. The structure with lowest energy was selected as the final full-length structure.

To validate the value of the full-length crystal structure (“crystal structure” always refers to the energy-minimised full-length structure after “RosettaCM” and “relax” protocols in the rest of the paper if not explicitly stated), the Rosetta “cartesian_ddg” [40] application (SI) was performed to calculate the △△*G* of variants upon point mutations and correlate with our previous *T*_m_ and aggregation rates [68]. The crystal model performed well in the correlation for both *T*_m_ and aggregation rates (*T*_m_: R^2^ = 0.95, ln(*v*): R^2^ = 0.50) (SI), giving broadly similar result as by the homology model [29]. Therefore, the crystal structure demonstrates a remarkable reflection for the structure.

### 4.2 Molecular dynamics simulations

Molecular dynamic (MD) simulations on the Fab A33 crystal model were conducted in Gromacs 2019.3 [69]. MD simulations were carried out at neutral pH and room temperature (pH 7.0 and 300 K) and under two stresses, low pH (pH 3.5 and pH 4.5 at 300 K) and high temperature (pH 7.0 at 340 K and 380 K). Many high temperature simulations are performed at relatively high temperatures (e.g. 500 K), to achieve complete denaturation of the protein. However, in this case, we aimed to partially unfold Fab A33 and detect the regions prone to early unfolding. Simulations were carried out using the OPLS-AA/L all-atom force field [70]. The Fab PDB file was first converted to a topology file with its five (four intra-chain and one inter-chain) disulfide bonds retained. The protonation state of each residue was entered manually, and these were determined at each pH using the PDB2PQR server, which performed the pKa calculations by PropKa [71]. This gave the following total charges: +7 (pH 7.0), +17 (pH 4.5) and + 31 (pH 3.5). The Fab A33 structure was centered in a cubic box with a layer of water up to at least 10.0 Å from the protein surface. The box was solvated with SPC/E water molecules, Cl^-^ added to neutralize the net charges, and NaCl added to an ionic strength of 50 mM for all simulations. The system was energy minimized using the steepest descent minimization integrator to achieve the maximum force less than 1000 kJ/mol/nm. The solvent and ions around the protein were equilibrated in position-restricted simulations for 100 ps under NVT ensemble to stabilize at the specified temperature, and then at 100 ps under NPT ensemble to stabilize at atmospheric pressure. Lastly, MD simulations were carried out for 100 ns in six replicates under the five conditions (pH 7.0 and 300 K; pH 4.5 and 300 K; pH 3.5 and 300 K; pH 7.0 and 340 K; pH 7.0 and 380 K). Jobs were submitted to the UCL Myriad High-Performance Computing Facility. The time step of the simulations was set to 2 fs and trajectories were saved every 100 ps.

### 4.3 Analysis of MD trajectories

MD trajectories were saved every 0.1 ns (total of 1001 frames). All-atom RMSD (side-chain included) of individual domains during the simulations were calculated using the Gromacs “gmx rms” tool, with corresponding domains as the reference. Domains were V_L_ (1–108), V_H_ (215–334), C_L_ (109 to 214) and C_H_1 (335 to 429). Averages and SEM of six independent repeats are shown. Radius of gyration (Rg) was calculated using the Gromacs “gmx gyrate” tool. Native contacts over simulation time were calculated using MDAnalysis [31], [32]. A soft cutoff distance was used in the calculations [33], [72] (SI). Fractions of native contacts were calculated for domain-level (Fig. 4) and residue-level (SI). Variable domain native contacts (V_L_-V_H_) were calculated between residues 1–108 (V_L_) and 215–334 (V_H_). Constant domain native contacts (C_L_-C_H_1) were calculated between residues 109–214 (C_L_) and 335–429 (C_H_1). Total contacts, including native and non-native ones, were calculated using the Gromacs “gmx mindist” tool (SI). RMSF of each domain was calculated using the Gromacs “gmx rmsf” tool. Both residue-level RMSF and native contact are projected on the protein structure (Fig. 5).

Secondary structure (SS) assignments of each residue along the trajectory were done using the DSSP module [53], [54]. For light chain, the variable domain is composed of 9 β-strands named A to I, while constant domain contains 7 strands named J to P; for heavy chain, the variable domain is composed of 10 β-strands named A to J, while constant domain has 8 β-strands named K to R (SI_Fig. 4). To calculate the loss in β-strand structure for each of the strands, we first tracked the secondary structure designation for each residue in Fab A33 throughout the simulations (SI). The percentage of time occupied within β-strand was calculated for each residue, and then summed for each of the 34 β-strands in Fab A33. This value was averaged for each of the six repeats at each condition. The percentage change in β-strand occupancy was then calculated, to determine the loss relative to the reference simulations at pH 7.0, 300 K.

Lastly, salt bridges were calculated along the trajectories using VMD and a cutoff distance between O and N groups of 3.2 Å. From these, the occurrence (%) of each salt bridge during the simulation was calculated, and averaged for the six independent repeats at each condition.

### 4.4 Mutational study and ΔΔG calculations by FoldX and Rosetta

The effect of mutations on the stability of Fab A33 was studied using FoldX[38], [39], [43] and the Rosetta method “cartesian_ddg”[41]. Both tools predicted the difference in folding free energy, ΔΔG, between the protein carrying a point mutation and the wildtype. Each of the 442 residues in the Fab A33 were mutated to the other 19 possibilities, totaling 8398 single mutants. For FoldX, the “RepairPDB” command was used first to energy minimize the structural model of Fab A33 until the energy converged, by rearranging the amino acid side chains. Next, the “BuildModel” command was used to introduce the point mutations, optimize the structure of the new protein variant, and calculate the stability change upon mutation. For Rosetta method “cartesian_ddg”, an example of mutation and option files, listing the parameters of the executable, can be seen in SI. Jobs were submitted to the UCL Myriad High-Performance Computing Facility.

### 4.5 Packing density

Occluded surface (OS) program was used to calculate the atomic packing of Fab A33 [36], [37]. The occluded surface packing (OSP) values are useful for identifying regions of loose packing in a protein. OSP values for each residue were calculated from the collection of extended normals (ray-lengths) that extend outward from the molecular surface until they intersect neighboring van der Waals surface. Analysis of these normals, their respective lengths and the surface area involved in the interaction, defines the packing of each atom in the protein.

### 4.6 Aggregation-prone regions (APR) predictions

Aggregation prone regions (APR) of Fab A33 were predicted using PASTA 2.0 [45], TANGO [46], AGGRESCAN [47] and MetAmyl [48], using the protein sequence as input. The regions in which three out of the four software packages identified an APR were selected, resulting in seven APRs. The Amylpred2 consensus tool was used to confirm the presence of these APRs [55]. To calculate the solvent accessible surface area (SASA) of each APR during the trajectories, the area per residue over the trajectory was calculated first, using gromacs analysis tool “sasa”, then summed for each APR. The SASA of APRs at pH 7.0 300 K were used as benchmarks to infer the influence of the other four stressed conditions.

### 4.7 Sequence entropy of Fab sequences

Fab homologous sequences were retrieved from the Protein Data Bank (PDB) [73] using the BLAST search tool, with “Mask Low Complexity”, 10.0 E-Value Cutoff and 70% sequence identity cutoff. The returned results were further filtered using the “Custom Report” tool, to retain only human and mouse species, including Homo sapiens, Mus and Rattus norvegicus. Homologous light and heavy chains were retrieved separately and limited to chains with 190–250 residues, totaling 1170 and 613 sequences, respectively. Only kappa (κ) light chains were returned as Fab A33 is the kappa type [27]. The sequences were then aligned using the “ClustalW Multiple alignment” tool [74] and consensus regions analysed by “Entropy H(x)” in BioEdit [60]. The maximum entropy is 3.04 for 20 possible amino acids plus a gap and minimum entropy is 0 representing a fully conserved residue. Sequence logos were created using WebLogo [57], [58] based on the entropy of Fab A33 homologous sequences.

## CRediT authorship contribution statement

**Cheng Zhang:** Investigation, Project administration. **Nuria Codina:** Investigation. **Jiazhi Tang:** . **Haoran Yu:** Supervision, Methodology. **Nesrine Chakroun:** Investigation. **Frank Kozielski:** . **Paul A. Dalby:** Conceptualization, Funding acquisition, Supervision, Writing - review & editing.
