# Supplementary material for: Comparison of the pH- and thermally-induced fluctuations of a therapeutic antibody Fab fragment by molecular dynamics simulation
Source: Comput Struct Biotechnol J. 2021 May 4;19:2726–41. doi: 10.1016/j.csbj.2021.05.005 (PMC8131956; doi:10.1016/j.csbj.2021.05.005)
Supplement: Supplementary data 1 [file mmc1.docx]

Supplementary Information for

Comparison of the thermal- and pH-induced fluctuations of a therapeutic antibody Fab fragment by molecular dynamics simulation

Cheng Zhang^1^, Nuria Codina^1^, Jiazhi Tang^2^, Haoran Yu^3^, Nesrine Chakroun^1^, Frank Kozielski^2^ and Paul A. Dalby^1, *^

^1^Department of Biochemical Engineering, University College London, Gordon Street, London, WC1E 7JE, UK

^2^Department of Pharmaceutical and Biological Chemistry, School of Pharmacy, University College London, 29–39 Brunswick Square, London WC1N 1AX, UK

^3^Department of Chemistry, University College London, 20 Gordon Street, London, WC1H 0AJ, UK

^*^Correspondence: [p.dalby@ucl.ac.uk](mailto:p.dalby@ucl.ac.uk)

# Fab A33 sequence

Fab A33 amino acid sequence separated by domains. The six CDRs in the V_L_ and V_H_ domains are highlighted in red.

V_L_

DIQMTQSPSSLSASVGDRVTITCKASQNVRTVVAWYQQKPGKAPKTLIYLASNRHTGVPSRFSGSGSGTDFTLTISSLQPEDFATYFCLQHWSYPLTFGQGTKVEIKR

C_L_

TVAAPSVFIFPPSDEQLKSGTASVVCLLNNFYPREAKVQWKVDNALQSGNSQESVTEQDSKDSTYSLSSTLTLSKADYEKHKVYACEVTHQGLSSPVTKSFNRGEC

V_H_

EVQLVESGGGLVQPGGSLRLSCAASGFAFSTYDMSWVRQAPGKGLEWVATISSGGSYTYYLDSVKGRFTISRDSSKNTLYLQMNSLRAEDTAVYYCAPTTVVPFAYWGQGTLVTVSSAST

C_H_1

KGPSVFPLAPSSKSTSGGTAALGCLVKDYFPEPVTVSWNSGALTSGVHTFPAVLQSSGLYSLSSVVTVPSSSLGTQTYICNVNHKPSNTKVDKKV

Hinge

EPKSCDKTHTSAA

# Residues involved in the interface between light and heavy chains in Fab A33

SI_Table 1 Residues involved in the interface between light and heavy chains in Fab A33

| V_L_ | A34, Y36, Q38, A43, P44, T46, Y49, H55, T85, F87, L89, H91, Y94, P95, L96, F98, Q100, G101 |
| --- | --- |
| V_H_ | V251, Q253, K257, G258, L259, E260, W261, T264, Y273, Y274, L275, D276, Y309, V316, P317, F318, A319, W321, G322, Q323 |
| C_L_ | F116, I117, F118, P119, S121, E123, Q124, S131, V133, L135, N137, N138, Q160, E161, S162, V163, T164, D167, S168, K169, T172, S174, L175, S176, T178, T180, V205, T206, K207, S208, F209, E213, C214 |
| C_H_1 | F340, P341, L342, A343, P344, S345, T353, A354, A355, L356, G357, L359, K361, S379, G380, H382, F384, P385, V387, Q389, S397, V399, T401, K427 |
| Hinge | K432, C434, D435, T437, T439, A441, A442 |

# RMSD and radius of gyration (Rg) of the whole protein during simulation

SI_Figure 1. RMSD and radius of gyration (Rg) of the whole protein with simulation time. A, B) RMSD of the whole protein with simulation time for different A) pHs and B) temperatures. C, D) Radius of gyration (Rg) of Fab A33 with simulation time for different C) pHs and D) temperatures. In all cases, the average of six independent simulations is shown with the SEM as error.

# RMSD for 400-ns simulation at 300 K

SI_Figure 2. RMSD of individual domains and the whole protein for 400-ns simulation at 300 K. A, B, C, D, I) RMSD for domain V_L_, V_H_, C_L_, C_H_1 and the whole protein, respectively, for pH 3.5, pH 4.5 and pH 7 at 300 K. E, F, G, H, J) Same as A, B, C, D, I, but without pH 4.5 condition. In all cases, the average of six independent simulations is shown with the SEM as error.

The 400-ns simulation reveals comparable results to their 100-ns counterparts. The simulations at 300 K were also continued at each pH, until they reached 400 ns. The C_L_ domain continued to deviate most significantly at low pH, confirming it as the most labile region. The V_L_ domain RSMD at low pH diverged further from that at pH 7, though only slightly, beyond the first 100 ns, suggesting its deformation occurred after that of the C_L_ domain. The V_H_ and C_H_1 domains diverged in RMSD initially, but then converged after 100 ns, implying the heavy chain was less susceptible to destabilisation at low pH.

# MD analysis of the native contacts from simulation trajectories

The native contact function^1,2^ is defined in SI_Equation 1, where *Q* is the fraction of native contacts, *r* is an array of contact distances at time *t*, *r*_0_ is an array of contact distances at time *t*=0 (reference distances), *β* is the softness of the switching function (default 5.0 Angstrom) , and *λ* is the reference distance tolerance (default 1.8, dimensionless).

| $Q\left( r,r_{0} \right)= \frac{1}{1+e^{\beta(r-\lambda r_{0})}}$ | SI_Equation 1 |
| --- | --- |

# RMSF of individual domains

See *rmsf_resi_1-108_VL.pdf, rmsf_resi_109-214_CL.pdf, rmsf_215-334_VH.pdf, rmsf_335-429_CH1.pdf*

# Total interface contacts including native and non-native ones

SI_Figure 3. Total interface contacts during simulation. Contacts with simulation time for different A, B) pHs and C, D) temperatures, values as labelled for the contacts numbers with 4 Angstrom cutoff, calculated by Gromacs "gmx mindist". In all cases, the average of six independent simulations is shown with the SEM as error.

# Secondary structure (SS) of each residue in Fab A33 with simulation time

Secondary structure (SS) of each residue in Fab A33 with simulation time, calculated using DSSP^3,4^. Representative SS evolution plots are shown for only one of the repeats at each condition, secondary structure type as indicated in the legend. Light chain ranges from Residue 1 to 214; heavy chain ranges from Residue 215 to 442.

SI_Figure 4. β-strand order shown by lettering (A-P) for light chain (A) and (A-R) for heavy chain (B)

| Chain ID | Domain ID | β-strand ID | Residue Range |
| --- | --- | --- | --- |
| Light Chain  (LC) | V_L_ | A | 4-7 |
|  |  | B | 10-13 |
|  |  | C | 19-25 |
|  |  | D | 33-38 |
|  |  | E | 45-50 |
|  |  | F | 62-65 |
|  |  | G | 70-75 |
|  |  | H | 84-90 |
|  |  | I | 102-106 |
|  | C_L_ | J | 114-118 |
|  |  | K | 129-139 |
|  |  | L | 145-150 |
|  |  | M | 159-163 |
|  |  | N | 173-182 |
|  |  | O | 191-197 |
|  |  | P | 205-210 |
| Heavy Chain  (HC) | V_H_ | A | 217-221 |
|  |  | B | 224-226 |
|  |  | C | 231-239 |
|  |  | D | 248-253 |
|  |  | E | 260-265 |
|  |  | F | 272-274 |
|  |  | G | 282-287 |
|  |  | H | 291-298 |
|  |  | I | 306-312 |
|  |  | J | 325-330 |
|  | C_H_1 | K | 338-342 |
|  |  | L | 353-363 |
|  |  | M | 368-372 |
|  |  | N | 381-383 |
|  |  | O | 387-389 |
|  |  | P | 392-403 |
|  |  | Q | 413-418 |
|  |  | R | 422-428 |

## pH 3.5, 300 K


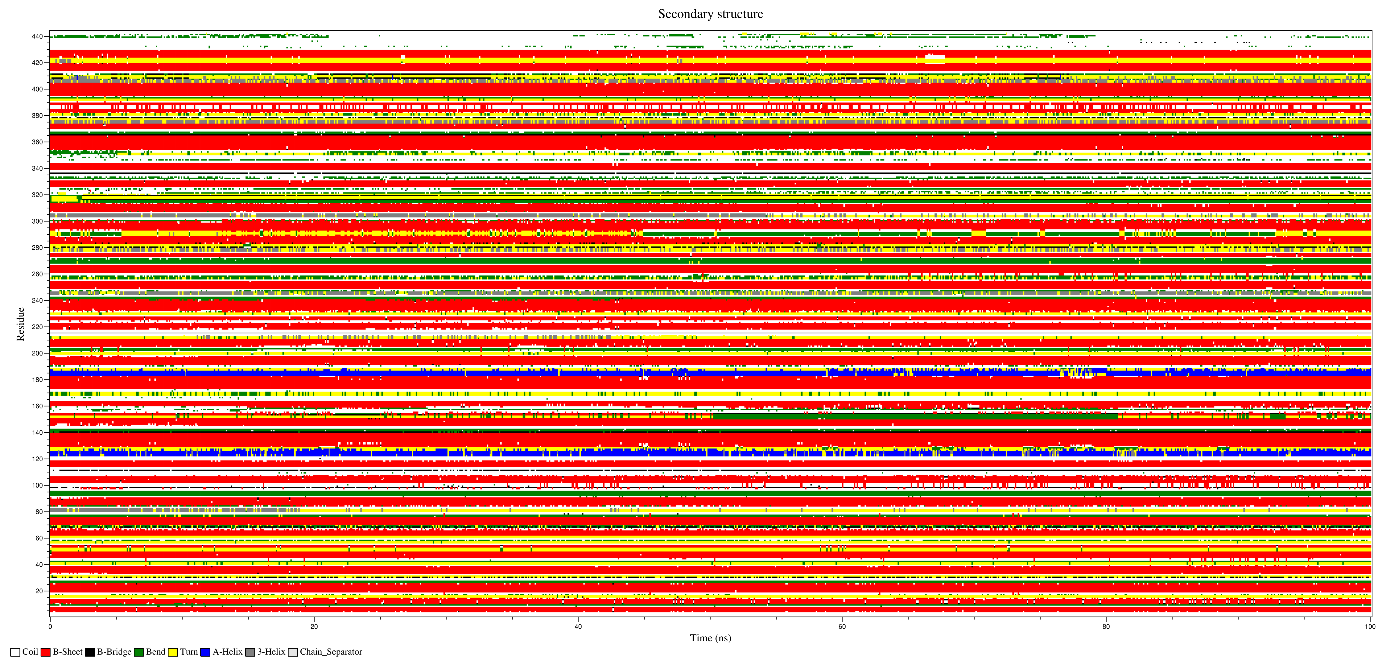


## pH 4.5, 300 K


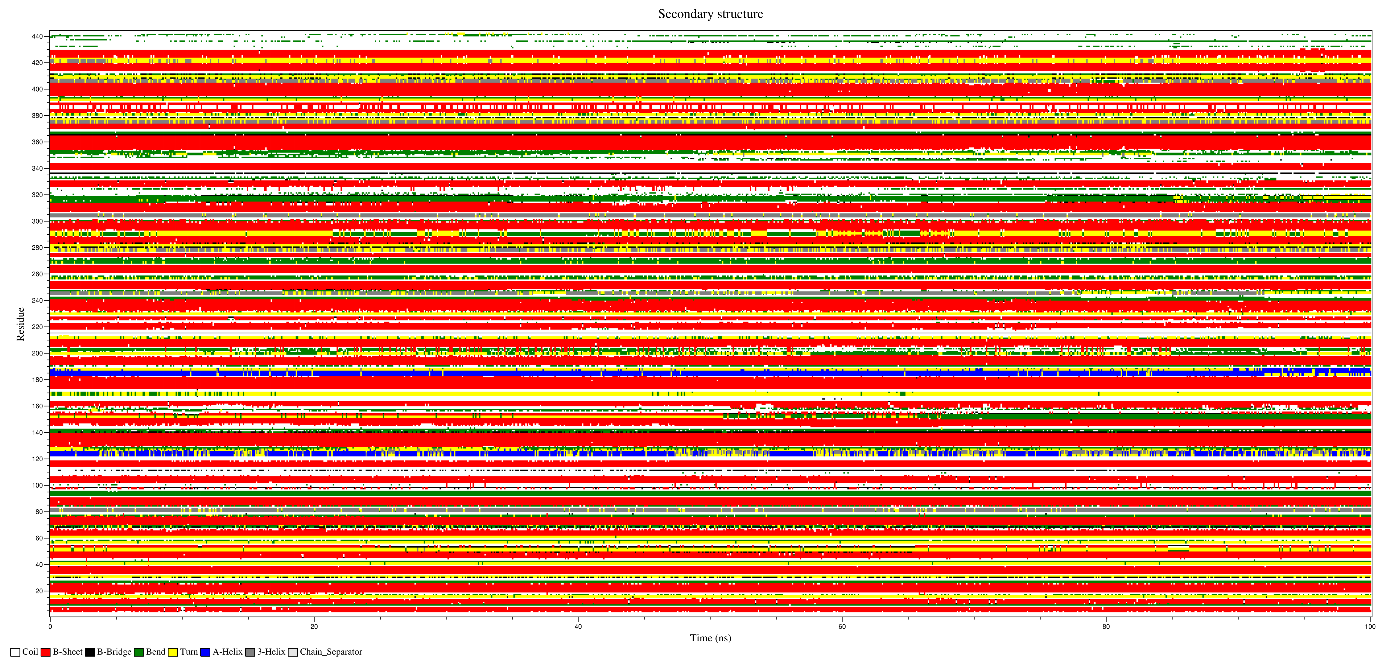


## pH 7.0, 300 K


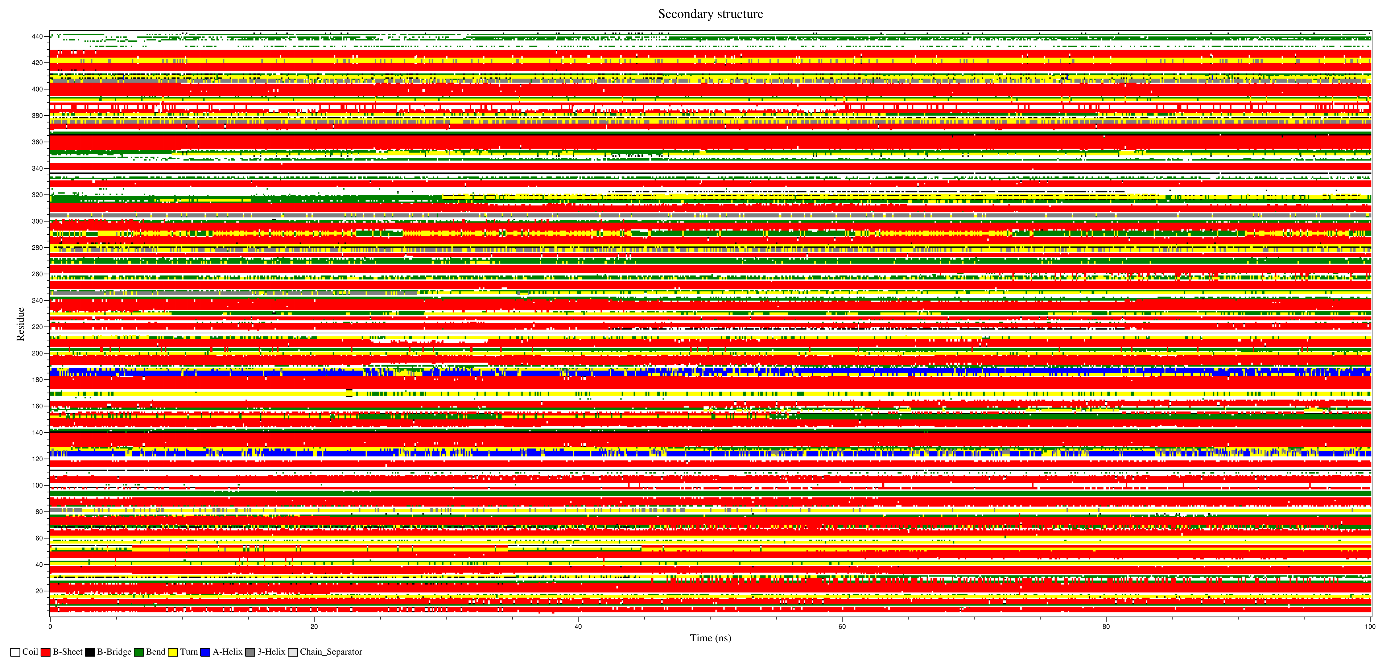


## pH 7.0, 340 K


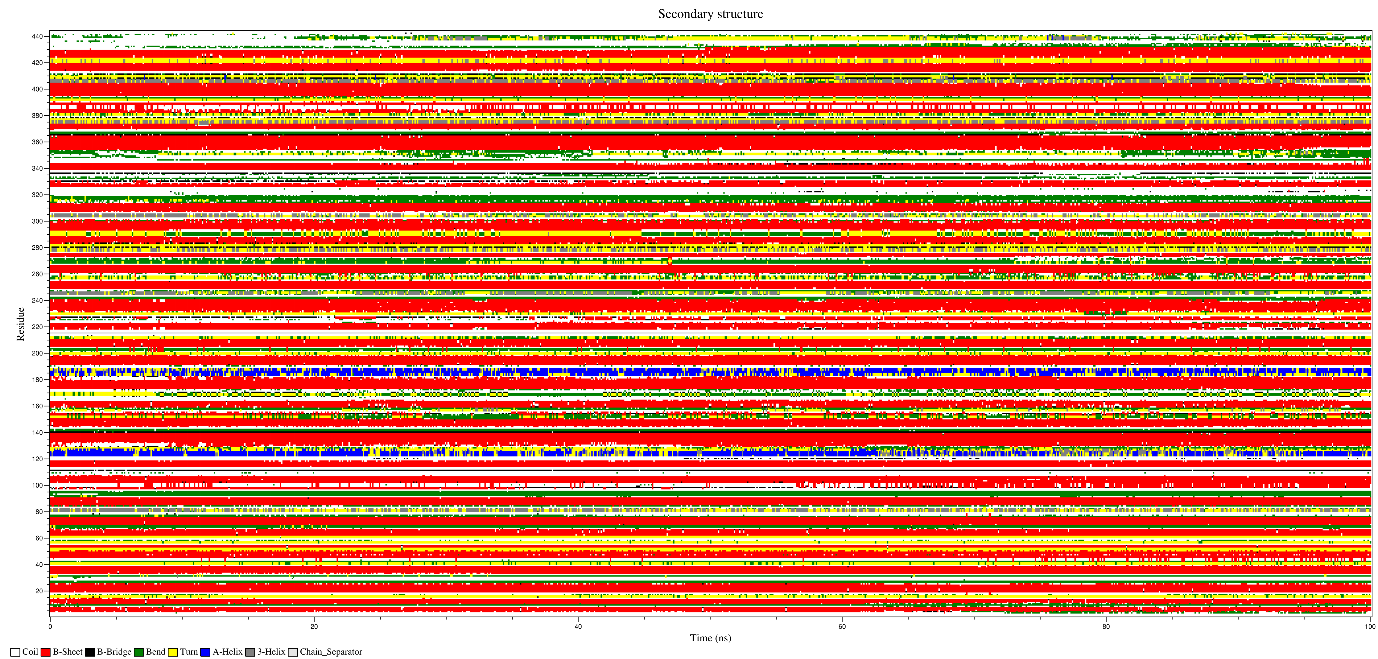


## pH 7.0 380 K


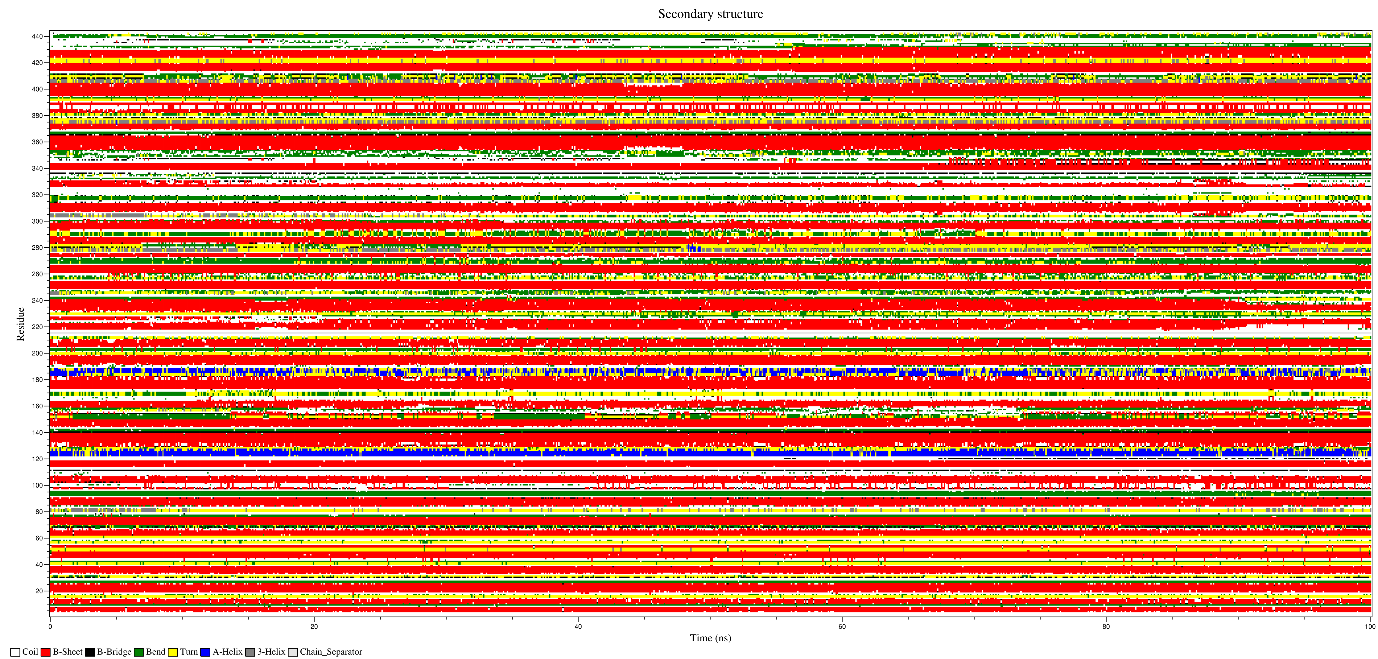


# Salt bridge analysis

pH 3.5, 300 K

(A)

(B)

pH 4.5, 300 K

(C)

(D)

pH 7.0, 300 K

(E)

(F)

pH 7.0, 340 K

(G)

(H)

pH 7.0 380 K

(I)

(J)

(K)

SI_Figure 5 Salt bridges formed during the simulation time using an O-N bond distance cutoff of 3.2 Å. For each condition, presence of a salt bridge is indicated in rainbow scale in the first figure (A, C, E, G, I). The overall averages of six independent simulations are shown in the second figure with their SEM as error (B, D, F, H, J). Occurrence of salt bridges during simulations, above a 5% threshold (K). The most persistent salt bridges are highlighted in the protein structures, coloured light grey and dark grey for light chain and heavy chain, respectively. Two critical salt bridges at low pH are highlighted in red dash circles for pH 7, 300 K. Values shown are the average of six independent simulations with error bars to show their SEM (K).

# Occluded Surface Packing


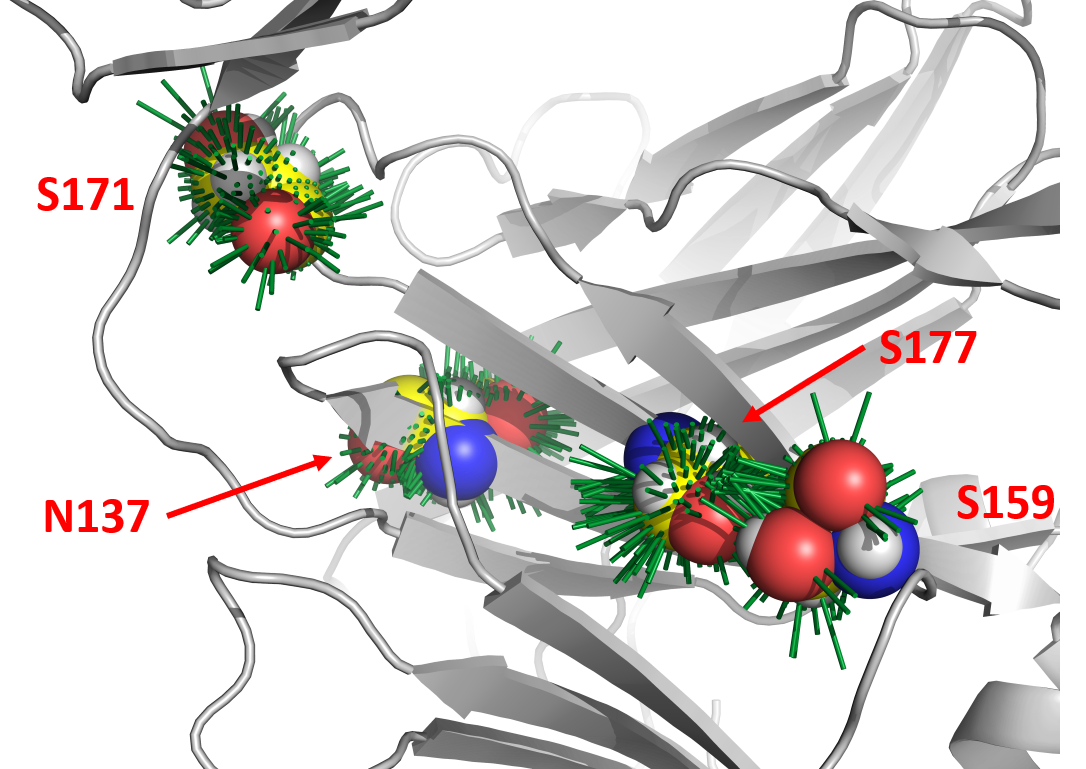


SI_Figure 6. Normals used to calculate the packing of each atom in Fab A33 using occluded surface software5,6. To calculate the occluded surface packing (OSP) value for each residue, normals that extend from the surface outward until they intersect a neighboring van der Waals surface were used. The normals used to calculate the OSP value are shown for the residues identified to have improved stability by FoldX and Rosetta.

SI_Table 2. The Occluded Surface Packing values for the β-stranded residues involved in the V_L_-V_H_ or C_L_-C_H_ interface contacts The average values are shown at the end with SEM.

SI_Figure 7. β-sheet residues not involved in the C_L_-C_H_1 interface contacts. The C_L_ and C_H_1 domains are in light and dark grey, respectively. The β-sheet residues not involved in the interface contacts (not in SI_Table 3) at the C_L_-C_H_1 interface are shown in red and magenta for C_L_ and C_H_1 domains, respectively.


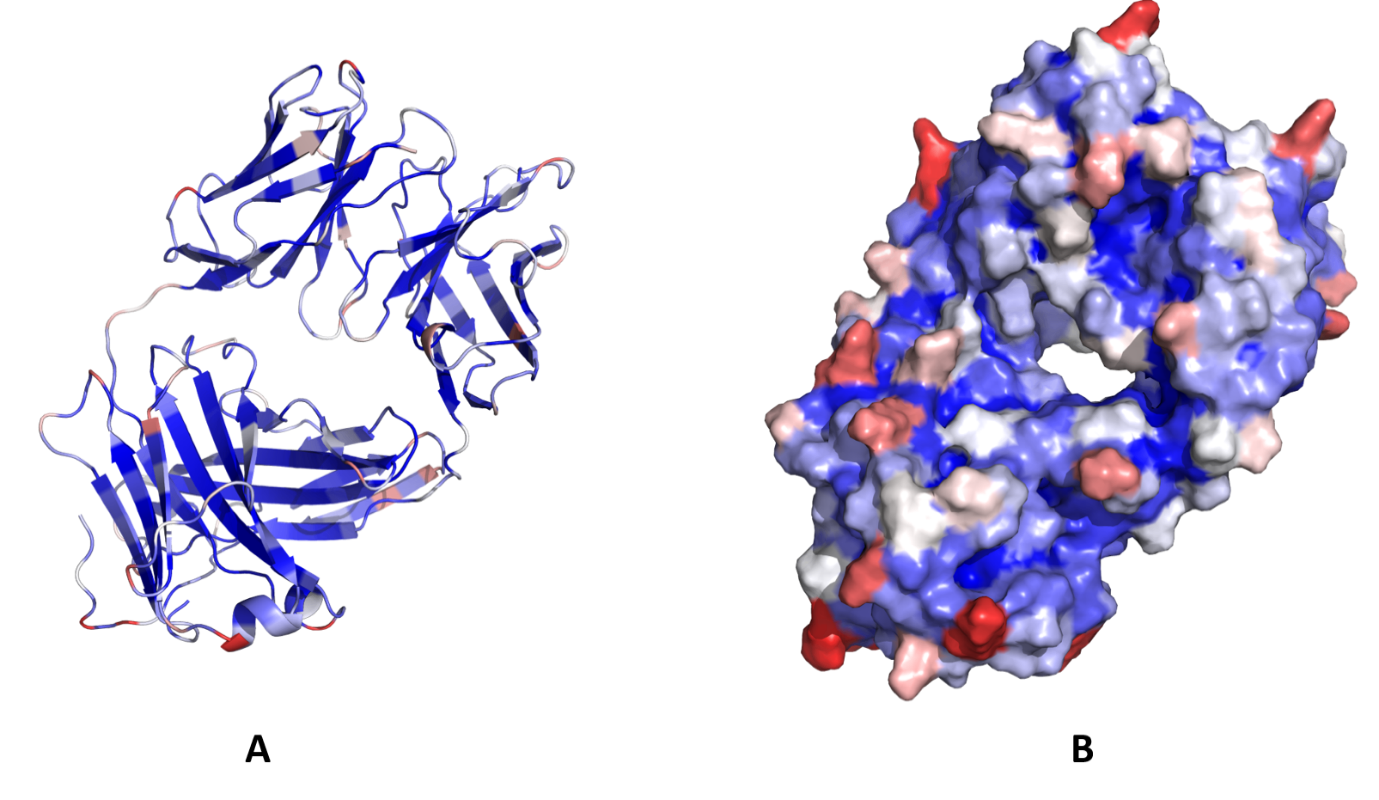


SI_Figure 8. The Solvent Accessible Surface Area (SASA) of the Fab crystal structure. The SASA was calculated by the Gromacs “sasa” command. Blue-white-red colour was assigned to the structure drawn by pymol for A) cartoon and B) surface.


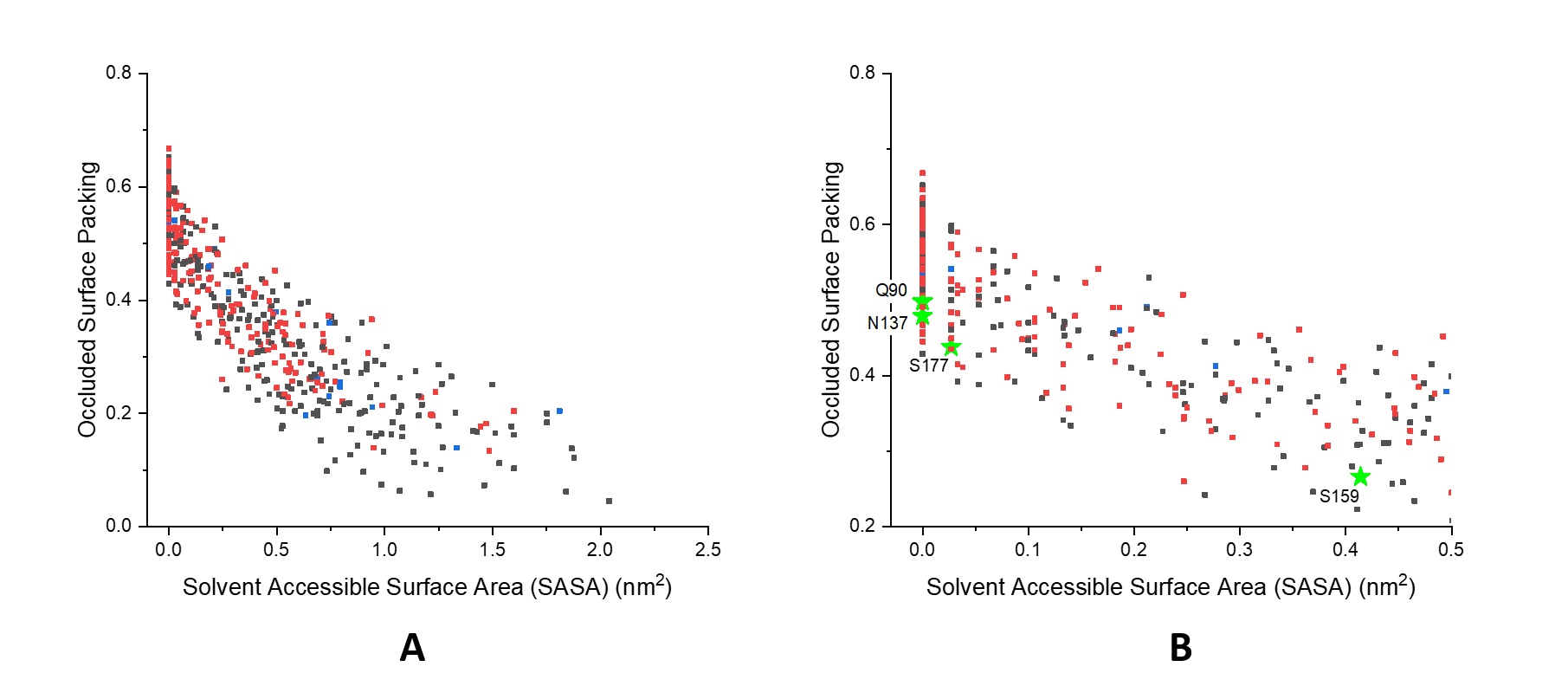


SI_Figure 9. The relation between the Occluded Surface Packing (OSP) and the Solvent Accessible Surface Area (SASA). The OSP and SASA were calculated for each residue, and coloured in black, red and blue for loop, sheet and helix structures, respectively. The Figure B) is amplified from Figure A) for the low SASA, high OSP region, where the under-packed residues were highlighted in green stars with labels.

# The command line for Rosetta

## relax

### relax.options

-in:fix_disulf Fab.disulfide

-in:file:s Fab.pdb

-nstruct 1000 # In reality, multiple jobs were submitted, totaling more than 20,000 structures

-in:file:fullatom

-out:file:fullatom

-out:pdb

-relax:quick

### Fab.disulfide

23 88

134 194

214 434

236 310

358 414

## cartesian_ddg

### cartesian_ddg.options

-s Fab.pdb

-ddg:iterations 3

-ddg::cartesian

-ddg::dump_pdbs true

-bbnbrs 1

-fa_max_dis 9.0

-score:weights ref2015_cart

-relax:cartesian

-relax:min_type lbfgs_armijo_nonmonotone

-ex1

-ex2

-use_input_sc

-flip_HNQ

-optimization:default_max_cycles 200

-crystal_refine

-ddg:mut_file A153P.mutfile

### A153P.mutfile (an example for single-point mutation)

total 1

1

A 153 P

# Comparison between Rosetta and FoldX


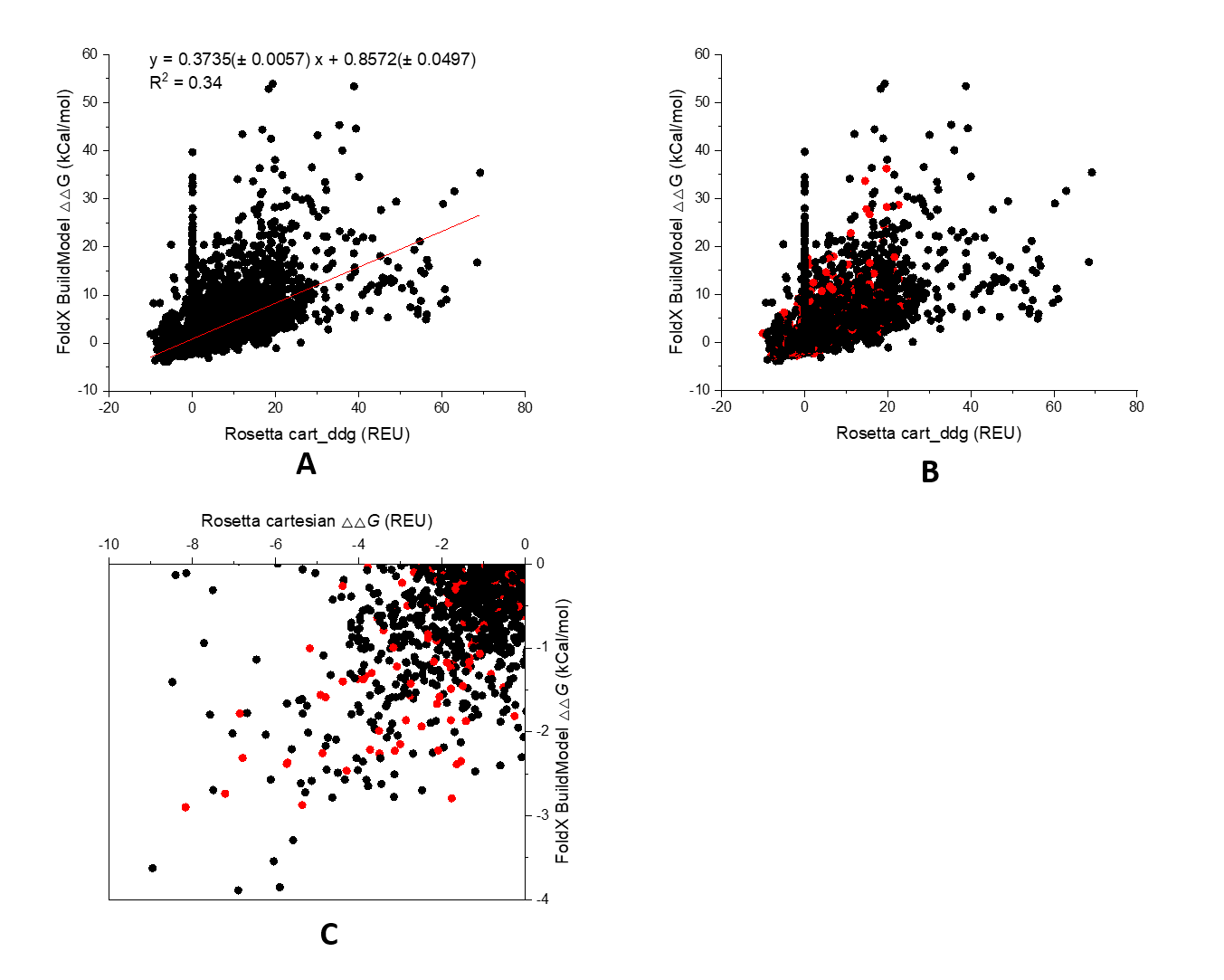


SI_Figure 10 FoldX and Rosetta predictions for the Fab variants. The “BuildModel” method was used for FoldX; the Cartesian_ddg was used for Rosetta. A) Correlation between FoldX and Rosetta predictions. B) The mutations involved in the interface contacts of V_L_-V_H_ and C_L_-C_H_1 domains were coloured in red, with C) showing only the third quadrant.

SI_Table 3. List of the most stabilizing mutations identified by FoldX^7–9^ BuildModel and Rosetta cartesian_ddg^10^. Mutation and ΔΔG of the 25 most stabilizing mutations predicted by FoldX and Rosetta.

| **Rosetta** | | **FoldX** | |
| --- | --- | --- | --- |
| **Mutant_ID** | **△△*G* (REU)** | **Mutant_ID** | **△△*G* (kcal/mol)** |
| S162Y | -10.08 | Q90L | -3.89 |
| S162F | -9.47 | Q90M | -3.85 |
| P420W | -9.37 | S177F | -3.62 |
| P420M | -9.14 | Q90I | -3.54 |
| S177F | -8.96 | S171F | -3.29 |
| P420A | -8.83 | T328Y | -3.15 |
| R30N | -8.65 | N137L | -2.90 |
| Q90F | -8.48 | S162M | -2.87 |
| K419A | -8.46 | S176M | -2.79 |
| R30G | -8.41 | G256R | -2.78 |
| N137L | -8.17 | Q90V | -2.77 |
| K419L | -8.15 | N137M | -2.74 |
| P420V | -8.04 | A254P | -2.72 |
| R30K | -7.97 | G256W | -2.70 |
| P420H | -7.82 | S177I | -2.69 |
| S159W | -7.72 | G256K | -2.65 |
| S177Y | -7.58 | S177M | -2.62 |
| P420N | -7.51 | S177L | -2.61 |
| R30D | -7.51 | T328I | -2.58 |
| S177I | -7.50 | S171M | -2.57 |
| P420Q | -7.38 | G336P | -2.57 |
| Q90Y | -7.26 | S395M | -2.57 |
| P420E | -7.22 | R142P | -2.51 |
| N137M | -7.21 | S395R | -2.49 |
| R211I | -7.15 | Q124L | -2.47 |

# Aggregation prone regions (APR)


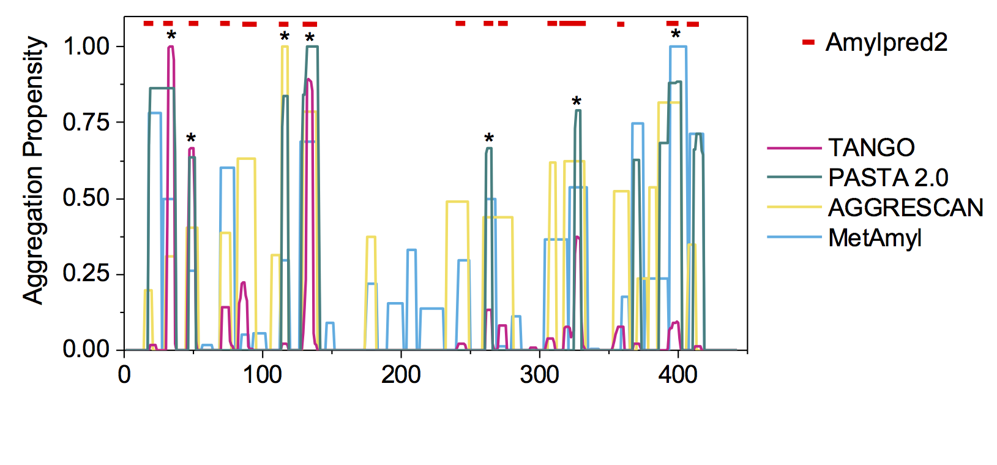


SI_Figure 11. Aggregation prone regions (APR) in Fab A33. A) The normalised aggregation propensity for each residue in Fab A33 was predicted using PASTA 2.0^11^, TANGO^12^, AGGRESCAN^13^ and MetAmyl^14^ software, each being colour-coded as shown in the legend. Aggregation-prone sequences where three of the four software agreed were selected, and highlighted with an asterisk. Amylpred 2^15^ consensus tool was also used to confirm the identification of those APRs, indicated in red on the top.

# Solvent Accessible Surface Area (SASA) of the Aggregation-Prone Regions (APR) in Fab A33 during simulation

SI_Table 4. SASA of the seven APRs in Fab A33 during simulations, shown in average values (nm^2^) ± SEM.

| APR residue range | Fab domain | pH3.5_300K | pH4.5_300K | pH7_300K | pH7_340K | pH7_380K |
| --- | --- | --- | --- | --- | --- | --- |
| 31-36 | V_L_ | 1.06 ± 0.07 | 0.87 ± 0.03 | 0.90 ± 0.04 | 0.84 ± 0.02 | 0.92 ± 0.05 |
| 47-51 | V_L_ | 1.31 ± 0.06 | 1.25 ± 0.02 | 1.24 ± 0.03 | 1.20 ± 0.04 | 1.27 ± 0.06 |
| 114-118 | C_L_ | 1.01 ± 0.08 | 1.09 ± 0.03 | 1.03 ± 0.05 | 1.08 ± 0.09 | 1.03 ± 0.05 |
| 129-139 | C_L_ | 1.56 ± 0.05 | 1.56 ± 0.07 | 1.44 ± 0.05 | 1.70 ± 0.07 | 1.62 ± 0.06 |
| 261-265 | V_H_ | 0.15 ± 0.02 | 0.12 ± 0.01 | 0.11 ± 0.00 | 0.19 ± 0.03 | 0.22 ± 0.03 |
| 325-329 | V_H_ | 0.84 ± 0.03 | 0.93 ± 0.04 | 0.98 ± 0.05 | 0.95 ± 0.06 | 0.96 ± 0.07 |
| 387-402 | C_H_1 | 6.02 ± 0.10 | 5.86 ± 0.14 | 5.80 ± 0.10 | 5.69 ± 0.06 | 5.54 ± 0.20 |

# The correlation between the △△*G* and *in vitro* stability data

SI_Figure 12 The correlation between the △△G and the in vitro stability data, T_m_ and aggregation rate ln(v). The Rosetta cartesian_ddg was used to calculate the △△G of variants upon point mutations for the full-residue crystal model. The T_m_ and aggregation rate are from previous work^16^.

# Relation between crystallographic B-factors and RMSF

We have a parallel manuscript on the crystallisation of the A33 Fab^17^. Two structures were resolved, namely a triclinic crystal (Space group P1) resolved at pH 9, and a hexagonal crystal (Space group P6_5_) resolved at pH 4. The P1 structure was firstly resolved, and was used for this MD paper.

The increased RMSF at 198-205 residues was not observed in the △B-factor. However, there was an increase of B-factor at residues 206-213. Residues 346-351 were not resolved in the crystal structures, so their B-factor values were not available.

SI_Figure 13. The relation between △b-factor (left Y-axis) and △RMSF (right Y-axis). The raw b-factor values for the atoms were averaged for each residue, for both the P1 and P6_5_ structures. The △b-factor values were calculated by subtracting the P1 b-factors from the P6_5_ ones. Positive △b-factors indicate increased b-factor in the P6_5_ (pH 4) compared to P1 (pH 9). The △RMSF values were from Fig 5A, C.

# Sequence logos of Fab A33 homologous sequences


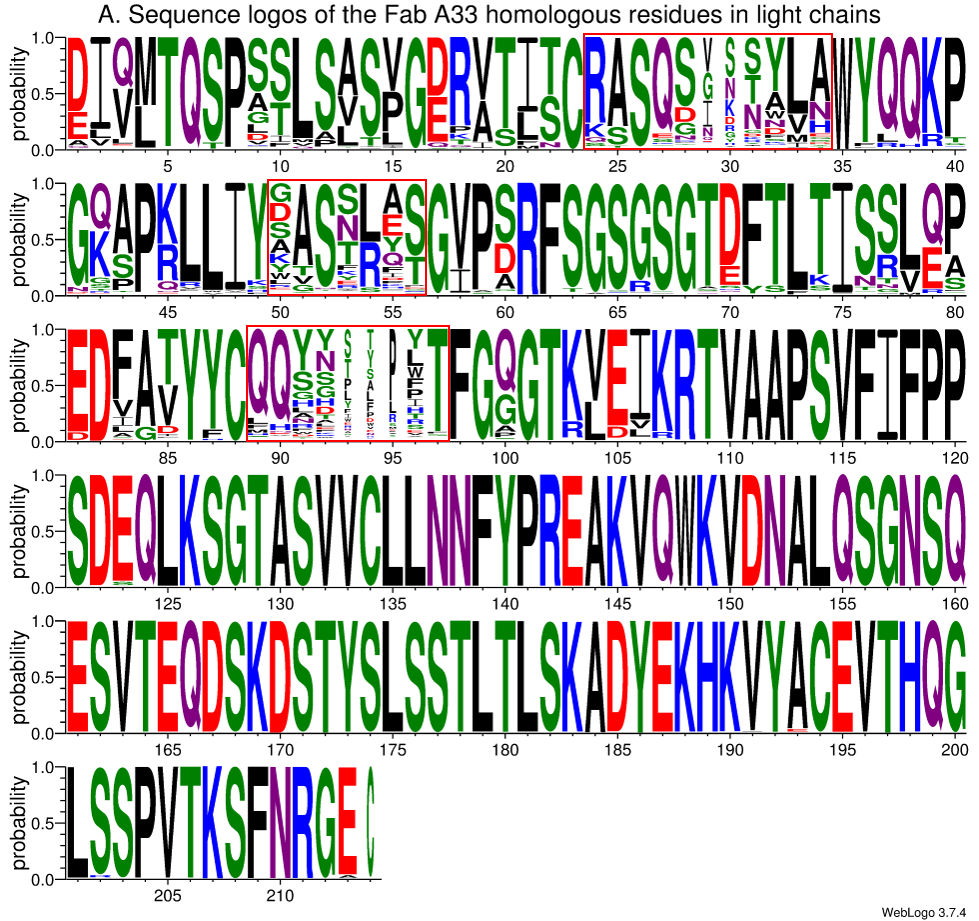


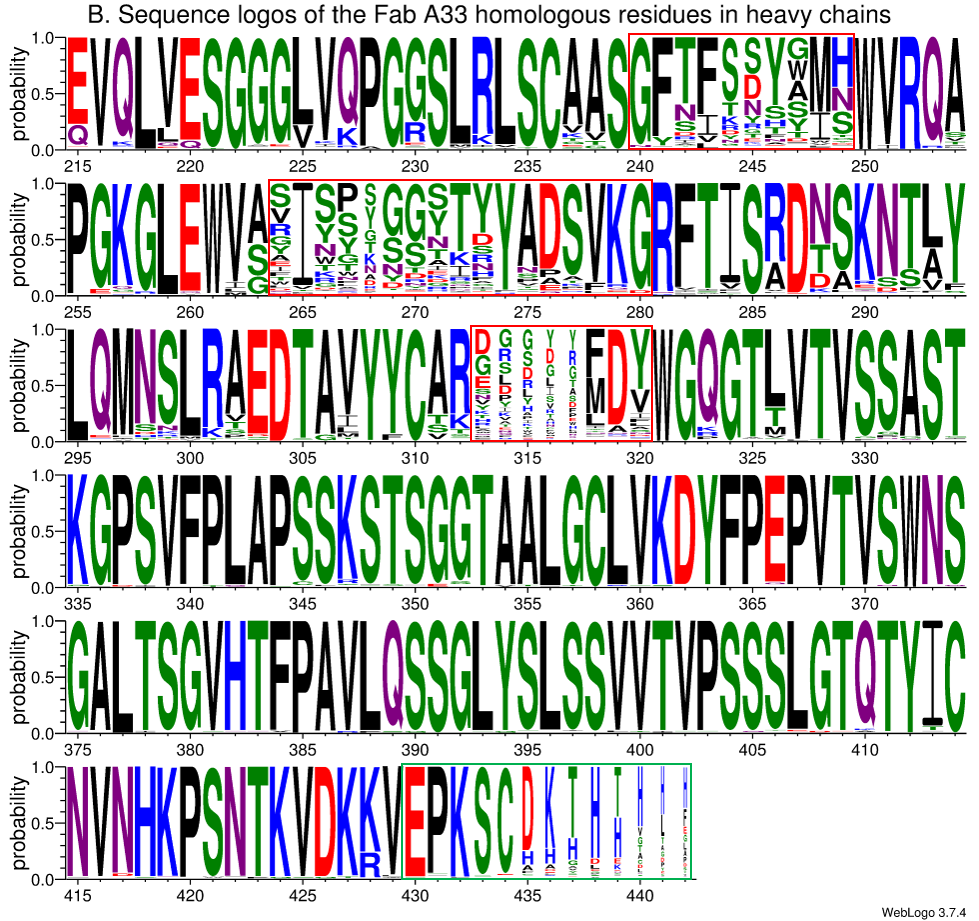


SI_Figure 14. The sequence logos of the Fab A33 homologous residues in light chains (A) and heavy chains (B). The position numbers correspond to the Fab A33 residue numbers. Logos were created with WebLogo^12,13^. Residues are coloured in green, purple, blue, red and black for polar, neutral, basic, acidic and hydrophobic types, respectively. Residues in CDR and hinge regions are boxed in red and green, respectively.

# Overview of the simulation parameters and analysis

| **Condition** | **Low pH** | pH 3.5, 50 mM, 300 K |
| --- | --- | --- |
|  |  | pH 4.5, 50 mM, 300 K |
|  | **Control** | pH 7.0, 50 mM, 300 K |
|  | **High temperature** | pH 7.0, 50 mM, 340 K |
|  |  | pH 7.0, 50 mM, 380 K |
| **Repeats per condition** | | 6 |
| **Sampling interval** | | 0.1 ns |
| **Simulation Time** | | 100 ns |
| **Analysis** | | Native interface contacts |
|  |  | Total interface contacts |
|  |  | RMSD (domain, overall) |
|  |  | RMSF (domain) |
|  |  | β-strands |
|  |  | Salt bridges |
|  |  | Packing Density (OSP) |
|  |  | ΔΔG: FoldX, Rosetta |
|  |  | SASA of APR |
|  |  | Sequence consensus |

# Supplementary References

1. Best, R. B., Hummer, G. & Eaton, W. A. Native contacts determine protein folding mechanisms in atomistic simulations. *Proc. Natl. Acad. Sci.* **110**, 17874–17879 (2013).

2. Beckstein, O., Denning, E. J., Perilla, J. R. & Woolf, T. B. Zipping and Unzipping of Adenylate Kinase: Atomistic Insights into the Ensemble of Open ↔ Closed Transitions. *J. Mol. Biol.* **394**, 160–176 (2009).

3. Touw, W. G. *et al.* A series of PDB-related databanks for everyday needs. *Nucleic Acids Res.* **43**, D364–D368 (2015).

4. Kabsch, W. & Sander, C. Dictionary of protein secondary structure: Pattern recognition of hydrogen‐bonded and geometrical features. *Biopolymers* **22**, 2577–2637 (1983).

5. Fleming, P. J. & Richards, F. M. Protein packing: Dependence on protein size, secondary structure and amino acid composition. *J. Mol. Biol.* **299**, 487–498 (2000).

6. Pattabiraman, N., Ward, K. B. & Fleming, P. J. Occluded molecular surface: Analysis of protein packing. *J. Mol. Recognit.* **8**, 334–344 (1995).

7. Guerois, R., Nielsen, J. E. & Serrano, L. Predicting changes in the stability of proteins and protein complexes: A study of more than 1000 mutations. *J. Mol. Biol.* **320**, 369–387 (2002).

8. Schymkowitz, J. *et al.* The FoldX web server: An online force field. *Nucleic Acids Res.* **33**, 382–388 (2005).

9. Buß, O., Rudat, J. & Ochsenreither, K. FoldX as Protein Engineering Tool: Better Than Random Based Approaches? *Comput. Struct. Biotechnol. J.* **16**, 25–33 (2018).

10. Park, H. *et al.* Simultaneous Optimization of Biomolecular Energy Functions on Features from Small Molecules and Macromolecules. *J. Chem. Theory Comput.* **12**, 6201–6212 (2016).

11. Walsh, I., Seno, F., Tosatto, S. C. E. & Trovato, A. PASTA 2.0: An improved server for protein aggregation prediction. *Nucleic Acids Res.* **42**, 301–307 (2014).

12. Fernandez-Escamilla, A.-M., Rousseau, F., Schymkowitz, J. & Serrano, L. Prediction of sequence-dependent and mutational effects on the aggregation of peptides and proteins. *Nat. Biotechnol.* **22**, 1302–6 (2004).

13. Conchillo-Solé, O. *et al.* AGGRESCAN: a server for the prediction and evaluation of ‘hot spots’ of aggregation in polypeptides. *BMC Bioinformatics* **8**, 65 (2007).

14. Emily, M., Talvas, A. & Delamarche, C. MetAmyl: A METa-predictor for AMYLoid proteins. *PLoS One* **8**, (2013).

15. Tsolis, A. C., Papandreou, N. C., Iconomidou, V. A. & Hamodrakas, S. J. A Consensus Method for the Prediction of ‘Aggregation-Prone’ Peptides in Globular Proteins. *PLoS One* **8**, 1–6 (2013).

16. Zhang, C. *et al.* Computational-design to reduce conformational flexibility and aggregation rates of an antibody Fab fragment. *Mol. Pharm.* **15**, 3079–3092 (2018).

17. Tang, J. & Kozielski, F. *Crystallization and synchrotron diffraction analysis of humanised A33 Fab, an immunotherapy candidate to colorectal cancer*. (2021).
